# Characterization of microbial community assembly in parasitic plant systems and the influence of microorganisms on metabolite accumulation in parasitic plants: case study of *Cistanche salsa* and *Kalidium foliatum*

**DOI:** 10.3389/fmicb.2024.1279536

**Published:** 2024-07-25

**Authors:** Zhan Feng, Yujing Miao, Xiao Sun, Yan Zheng, Guangming Luo, Jin Pei, Linfang Huang

**Affiliations:** ^1^Key Laboratory of Chinese Medicine Resources Conservation, State Administration of Traditional Chinese Medicine of the People’s Republic of China, Institute of Medicinal Plant Development, Chinese Academy of Medical Sciences & Peking Union Medical College, Beijing, China; ^2^State Key Laboratory of Southwestern Chinese Medicine Resources, College of Pharmacy, Chengdu University of Traditional Chinese Medicine, Chengdu, China; ^3^Jiangxi University of Chinese Medicine, Nanchang, China

**Keywords:** *Cistanche salsa*, *Kalidium foliatum*, microbiome, metabolomics, parasitic plants

## Abstract

**Introduction:**

*Cistanche salsa* (C.A.Mey.) G. Beck is a perennial holoparasitic herb recognized for its medicinal properties, particularly in kidney-tonifying and laxative treatments. Despite its therapeutic potential, little is known about the endophyte communities inhabiting *C. salsa* and its host plants, and how these microorganisms may impact the production and accumulation of metabolites in *C. salsa*.

**Methods:**

We conducted a dual analysis focusing on metabolomics of wild *C. salsa* and microbiome characterization of both *C. salsa* and its host plant, *Kalidium foliatum* (Pall.) Moq. The metabolomics analysis revealed variations in metabolite composition across different parts of *C. salsa*. Additionally, the microbiome analysis involved studying endophytic bacteria and fungi, comparing their community structures between parasitic *C. salsa* and its host plant.

**Results:**

Significant variations in metabolite composition were observed through metabolomic profiling, which identified 93 secondary metabolites and 398 primary metabolites across various parts of *C. salsa*. Emphasis was placed on differences in metabolite composition within the flowers. Microbiome analysis revealed differential community compositions of endophytic bacteria between the parasitic and host plants, whereas differences in endophytic fungi were less pronounced. Certain endophytes, such as Bacteroidota, Proteobacteria, Ascomycota, and Basidiomycota, were associated with the production of specific secondary metabolites in *C. salsa*, including the plant-specific compound salsaside.

**Discussion:**

Our findings highlight the intricate relationship between *C. salsa* and its endophytic microbiota, suggesting a potential role of these microorganisms in modulating the biosynthesis of bioactive compounds. The differential preferences of endophytic bacteria and fungi across various microenvironments within the parasitic plant system underscore the complexity of these interactions. Further elucidation of these dynamics could enhance our understanding of *C. salsa’s* medicinal properties and its ecological adaptations as a holoparasitic herb.

## 1 Introduction

*Cistanche salsa* (C.A. Mey.) G. Beck is a perennial parasitic herb belonging to the genus *Cistanche* Hoffmanns. & Link in the family Orobanchaceae Vent. It predominantly parasitizes plants from the genera *Kalidium* Moq., *Nitraria* L., and *Suaeda*, which are widely distributed in Inner Mongolia, Ningxia, Gansu, Qinghai, and Xinjiang, China (Wang, 1990). The traditional Chinese medicine standard of Gansu Province includes *C. salsa* as one of the original plants used in Chinese herb Cistanches (Administration, G.P.F.a.D, 2009), and possesses various medicinal properties, including antioxidant, anti-aging, laxative, and hepatoprotective effect (Chen et al., 2012). However, studies on this species remain limited, and due to over-harvesting and difficulties in artificial cultivation in recent years, the demand for this medicinal plant exceeds the available resources, leading to near depletion of medicinal resources.

The ecological niche shift of plants is a significant transition in their role and function in an ecosystem, often involving a shift from autotrophy to heterotrophy. While autotrophs obtain energy and nutrients through photosynthesis and root uptake, heterotrophs rely on interactions with other organisms to acquire the nutrients they need. Special organs like the haustorium allow heterotrophs to absorb nutrients from host plants by connecting with their roots or stems (Joel, 2013). Additionally, symbiotic mycorrhizal fungi can provide nutrients to plants and form a mutually beneficial relationship with their root systems (Spallek et al., 2017). Approximately 1–2% of flowering plants are directly parasitic on other plants to obtain carbon, water, and minerals (Westwood et al., 2010). Holoparasites are a type of heterotroph that are entirely reliant on host plants for their survival. This ecological niche shift is often accompanied by significant ecological and physiological changes, including the loss of photosynthetic functions and the development of morphological and genomic alterations such as the evolution of mutilated, scale-like leaves, the loss of a well-developed root system, and a reduction in chloroplast genome size. This phenomenon is collectively referred to as “parasitism reduction syndrome” (Colwell, 1996).

Plant metabolites are a group of compounds that are produced as intermediate and final products during metabolic processes within plant cells (Deng et al., 2020). Most studies on *C. salsa* have focused on identifying its main active substances, including phenylethanol glycosides, cyclic enol ether terpenoids, polysaccharides, and alkaloids (Lei et al., 2020). However, it is imperative to note that these components constitute merely a fraction of the pharmacologically active substances present within *C. salsa*. Studies have shown that each plant is composed of thousands of compounds and that many as yet unidentified trace compounds also affect the overall pharmacological activity (Lakshminarasimman et al., 2018). Therefore, it is necessary to systematically investigate and characterize the complete metabolic profile of *C. salsa*.

Plants harbor diverse microbial communities that vary in diversity and composition based on host plant tissues, individuals, and species (Bulgarelli et al., 2013). These differences are influenced by environmental factors such as the soil indigenous microbiome (Liu et al., 2019), plant innate immunity (Stringlis et al., 2018), quality and quantity of plant-derived resources (Zhalnina et al., 2018), and interactions between microorganisms (Carlström et al., 2019). Control experiments have demonstrated that microbiota play a critical role in nutrient acquisition by plants (Finkel et al., 2019), tolerance to abiotic and biotic stress (Fitzpatrick et al., 2018), and defense against pathogens (Tsolakidou et al., 2019). Our research group has previously studied wild *C. salsa* in diverse habitats (Sun et al., 2020), employing a combination of the MaxEnt model and 16S rRNA amplicon sequencing, the researchers aimed to determine the optimal growth conditions and locations for *C. salsa* while also identifying microbial markers in soils from various habitats. Additionally, we performed microbial sequencing analysis on *Cistanche deserticola* and its host plant *Haloxylon ammodendron* (Miao et al., 2023a), elucidating a striking resemblance in the root microbiota between the parasitic plant and its host. In light of these findings, we introduced the notion of “parasitic equilibrium.” Subsequently, comparing the microbiome of the rhizosphere of both plant species it was shown the complexity of the rhizosphere microbiome of the host plant was reduced after parasitism. Furthermore, we discerned the presence of bidirectional microbial communication between the parasitic plant and its host (Miao et al., 2023b). However, differences in microbiota composition exist between host plant species, but the causes and ecological consequences of this variation are not fully understood (Fitzpatrick et al., 2018). To better comprehend the composition and function of plant microbiota, it is necessary to investigate plant species occupying distinct ecological niches (Coleman-Derr et al., 2016). This will allow for an exploration of the formation of associated microbiota and the identification of functional diversity among different plant species.

Presently, there remain research gaps concerning *C. salsa*, encompassing the following inquiries: (1) What constitutes the overall metabolic profile of *C. salsa*, and are discrepancies apparent in the metabolite profiles across its various components? (2) Does a disparity exist in the composition of the endophytic microbial community between *C. salsa* and its host plants? (3) Is there a plausible correlation between metabolite distinctions across different components of *C. salsa* and its endosymbiotic bacteria? To address these questions, this study used *C. salsa* and its host plant *Kalidium foliatum* (Pall.) Moq. for the following analyses: (1) Ultra-performance liquid chromatography-quadrupole time-of-flight mass spectrometry (UPLC-Q-TOF-MS) was used to analyze the powders of different parts of *C. salsa* to identify metabolic differences among different parts; (2) Amplicon sequencing of microbial communities of different parts of *C. salsa* and *K. foliatum* as well as the rhizosphere was performed to explore the distribution characteristics of microorganisms in the whole parasitic plant system; (3) correlation analysis of differentially enriched metabolites of different parts of *C. salsa* with endophytes was carried out to identify endophytes that affect metabolite accumulation.

## 2 Materials and methods

### 2.1 Sample information

**Figure 1:**
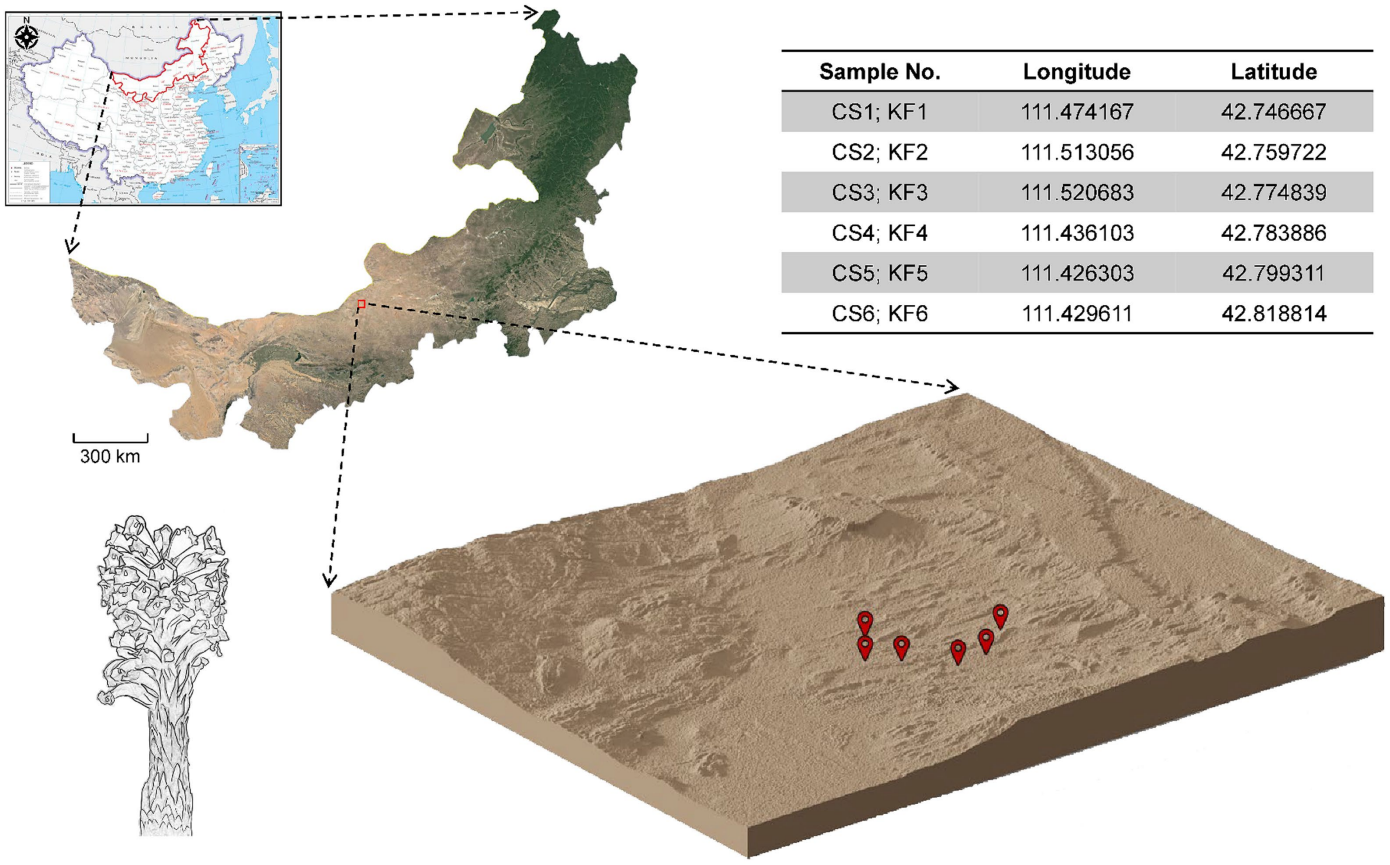
Collection sites of *C. salsa* and *K. foliatum*. Plant samples were collected from Siziwangqi, Inner Mongolia, China. The table in the upper right corner provides latitude and longitude coordinates for each sample group’s collection site. The three-dimensional map illustrates the topography of the collection site, with red markers indicating the sampling locations.

In March 2021, samples of *C. salsa* and *K. foliatum* were collected as whole plants in Siziwangqi, Inner Mongolia, China (Figure 1). The roots of individual flowering *C. salsa* plants were excavated with precision and connected to their respective host plant roots. Rhizosphere sampling adhered to the protocol followed by Edwards (Edwards et al., 2015). Excess soil adhering to the roots was manually removed, leaving approximately 1 mm of soil still affixed. Subsequently, the roots were carefully placed in a sterile flask containing 50 mL of sterile phosphate buffered saline (PBS) solution. To ensure thorough soil removal from the root surface, the roots were vigorously agitated using sterile forceps. The soil extracted from the roots was transferred to 50 mL Falcon tubes and designated as rhizosphere material. Six biological duplicates were collected for each sample type. All specimens were promptly immersed in liquid nitrogen, then transported on dry ice to the laboratory in Beijing, and finally preserved at −80°C until subsequent processing. The samples were identified as *Cistanche salsa* (C.A. Mey.) G. Beck and *Kalidium foliatum* (Pall.) Moq by researcher Linfang Huang of the Institute of Medicinal Plants, Chinese Academy of Medical Sciences, and voucher specimens were stored in the Herbarium of the same institute with the codes CMPB16301 and CMPB16302.

### 2.2 Metabolome analysis

#### 2.2.1 Preparation of the samples

The flowers, leaves, and succulent stems of *C. salsa* were excised using a sterile blade, ensuring the selection of healthy tissue. Subsequently, the excised portions underwent surface sterilization: first with a 2% solution of sodium hypochlorite (containing 0.1% active chlorine) for one minute, followed by exposure to 75% ethanol for 30 s. Each sample collection was performed with a freshly cleaned blade, swabbed with 70% ethanol after each use. Following this, the samples were subjected to a triple wash with sterile distilled water, followed by air-drying on sterile filter paper under aseptic conditions. The dried samples were then lyophilized, crushed, and passed through a No. 3 sieve.

Subsequently, 100 mg of sample powder was dissolved in 1.2 mL of a 70% methanol solution and vortexed for 30 s at 30 min intervals, repeating this process six times in total. The samples were then subjected to centrifugation at 12,000 rpm for 10 min. The resulting supernatant was subsequently filtered through a 0.22 μm microporous membrane prior to storage in injection vials for subsequent UPLC-Q-TOF-MS analysis.

#### 2.2.2 UPLC-Q-TOF-MS analysis

The column temperature was maintained at 25°C, with a flow rate of 0.30 mL/min and an injection volume of 2 μL. Mobile phase A consisted of 0.1% formic acid water, while mobile phase B consisted of acetonitrile. Gradient elution was used with the following conditions: 0–3 min, 10–22% B; 3–4 min, 22–23% B; 4–6 min, 23–35% B; 6–8 min, 35–37% B; 8–11 min, 37–42% B; 11–12 min, 42–48% B; and 12–15 min, 48–10% B. The electrospray ionization ion source was used with positive ion mode detection, with a cone hole voltage range of 4–35 kV, an ion source temperature of 100°C, a desolvation temperature of 450°C, and nitrogen gas used for both atomization and desolvation, with a volume flow rate of 800 L/h. Collision gas was Ar, with a collision pressure of 7.066 mPa, and the mass-to-charge ratio was corrected to m/z 556.2771. The scan range was set at m/z 100 to 1,200, and data were analyzed using Masslynx V4.1 (Waters).

#### 2.2.3 Data analysis

Prior to pattern recognition, the raw data require pre-processing. Baseline filtering, peak identification, integration, retention time correction, peak alignment, and normalization were carried out with Progenesis QI v3.0. Precise mass numbers, secondary fragmentation, and isotopic distribution were combined with the PMDB database (Luming Biological Technology Co. Ltd., Shanghai, China) and public databases to qualitatively analyze the compounds. The extracted data underwent screening based on the qualitative result score (Score), with screening criteria retaining data with Score > 36 (out of a total score of 60). These criteria encompass exact molecular weight matches for primary mass spectra (20), fragment matches for secondary mass spectra (20), and isotopic distribution matches (20). Generally, higher scores indicate more precise characterization results. Compounds with Score < 36 were deemed inaccurate and subsequently excluded. OmicShare Tools was used to perform Principal components analysis (PCA) and Orthogonal partial least-squares discrimination analysis (OPLS-DA). The Variable important in projection (VIP), log2 Fold Change (FC) values and *p*-values were obtained by orthogonalizing the X variables in the OPLS-DA analysis. Metabolites were considered as differentially enriched metabolites when they satisfied both VIP value >1, *p*-value value <0.05 and |log2 (FC)| > =1 (Zakharov et al., 2019). Finally, metabolic pathway enrichment analysis was performed using the Kyoto Encyclopedia of Genes and Genomes database (KEGG) database to investigate the biological functions of the differentially enriched metabolites (Kanehisa et al., 2023).

### 2.3 Microbiological analysis

#### 2.3.1 Plant and soil sampling

Plant samples were collected from each sampling site and immediately transported to the laboratory for DNA extraction of endophytes (Lundberg et al., 2012). The parasitic plant *C. salsa* was sampled for flower (CSF), scale leaf (CSL), fleshy stem (CSS), haustorium (H) and rhizosphere (KFRS), while the host *K. foliatum* was sampled for flower (KFF), stem (KFS), root (KFR) and rhizosphere (KFRS). Multiple scale leaves and flowers were sampled from each parasitic plant individual, and at least three samples were collected from each *K. foliatum* individual. For the sterilization method of plant surfaces, please refer to Section 2.2.1.

The plant samples were placed in a 50 mL centrifuge tube containing 25 mL sterile phosphate buffer, vortexed for 15 s, and then transferred to a new 15 mL centrifuge tube containing 5 mL sterile buffer. This process was repeated twice. After the final vortex, the sample was sonicated at low frequency for 5 min (5 groups of 30 s with 30 s intervals) in a 1.7 mL centrifuge tube containing 1 mL of sterile buffer. To extract DNA, the samples were ground in each tube with two 5 mm diameter steel beads for 2 min due to the physical toughness of plant tissues. The rhizosphere soil was concentrated by pipetting 1 mL of the PBS/rhizosphere soil into a 2 mL tube and centrifuging for 30 s at 10,000 g. The supernatant was discarded leaving only the soil fraction behind.

#### 2.3.2 PCR amplification and sequencing

DNA was extracted from both plant and soil samples using a DNA extraction kit (E.Z.N.A. Soil DNA Kit, Omega Bio-tek, United States). The quality and concentration of DNA were evaluated using an ultra-micro spectrophotometer (Nanodrop 2000, ThermoFisher, Inc., United States). Using primers (ACTCCTACGGAGGCAGCAG) and (GGACTACHVGGGTWTCTAAT) to amplify the bacterial 16S rRNA V3–V4 region, and primers (CTTGGTCATTTAGAG GAAGTAA) and (CTTGGTCATTTAGAGGAAGTAA) to amplify the fungal ITS1 region. An 8 bp barcode sequence was added at the 5′ end of each upstream and downstream primer to distinguish between the different samples. Subsequently, the universal primers with barcode sequences were amplified on a PCR instrument, and each sample was replicated three times. The PCR reaction system had a total volume of 25 μL, including 2 μL of DNA template (30 ng of DNA added), 1 μL of Forward Primer (5 μM), 1 μL of Reverse Primer (5 μM), 3 μL of BSA (2 ng/μL), 12.5 μL of 2 × Taq Plus Master Mix (Vazyme, China), and 5.5 μL of ddH2O. The PCR amplification protocol consisted of pre-denaturation at 95°C for 5 min, denaturation at 95°C for 45 s, annealing at 55°C for 50 s, extension at 72°C for 45 s, 30 cycles for bacteria and 34 cycles for fungi, and final extension at 72°C for 10 min. After mixing the three PCR amplification products well, the size of the amplification products was detected using 1% agarose gel electrophoresis and then purified using a nucleic acid purification kit (Agencourt AMPure XP, Beckman Coulter, United States). Subsequently, library construction of PCR products was performed using a library construction kit (NEB Next Ultra II DNA Library Prep Kit, New England Biolabs, Inc., United States) and purified using the nucleic acid purification kit. The library concentration was initially measured using an ultra-micro spectrophotometer (Nanodrop 2000, ThermoFisher, Inc., United States), and the size of the library fragments was determined using a bioanalyzer (Agilent 2,100 Bioanalyzer, Agilent Technologies, Inc., United States) to ensure that the constructed libraries met the specifications. The precise concentration of the library was determined using ABI StepOnePlus Real-Time PCR System (Applied Biosystems, Inc., United States). After determining the library concentration, the libraries were sequenced using a second-generation sequencer (Miseq PE300, Illumina, Inc., United States).

#### 2.3.3 Data processing and analysis

##### 2.3.3.1 Data allegation and OTUs cluster analysis

First, the Pair-End (PE) sequence data obtained from Miseq sequencing was assigned to different samples based on their respective barcode sequences using the QIIME (v1.8.0) (Caporaso et al., 2010). Next, the sequencing data underwent quality filtering and splicing using the Pear (v0.9.6) (Zhang et al., 2014). Filtering was done to remove sequences with ambiguous bases, primer mismatches, and those that scored below 20. The minimum overlap for splicing was set at 10 bp with a mismatch rate of 0.1. Vsearch (v2.7.1) (Rognes et al., 2016) was used to remove bacterial sequences less than 230 bp in length after splicing and chimeric sequences were removed according to the UCHIME method using the Gold Database (Edgar et al., 2011). Similarly, for fungal ITS1, Vsearch (v2.7.1) was used to remove sequences less than 120 bp in length (230 bp for ITS2) after splicing and chimeric sequences were removed according to the Unite Database using the UCHIME method. The high-quality sequences were subjected to OTU clustering using the UPARSE (Edgar, 2013) algorithm in Vsearch (v2.7.1). For ITS amplicon sequencing, the sequencing depth was 32,080 tags per sample. In 16S amplicon sequencing, a step was incorporated to eliminate plant chloroplast and mitochondrial sequences. Initially, the standard procedure of OTU clustering and annotation was executed. Subsequently, based on the results of OTU annotations, those annotated to mitochondria and chloroplasts were excluded. Downstream analyses were conducted employing the OTUs remaining after the removal of mitochondria and chloroplasts. The sequencing depth amounted to 22,061 tags per sample prior to removal and 118 tags per sample thereafter. Clustering was done according to different similarity levels and bioinformatic statistical analysis was carried out for OTUs at the 97% similarity level. The OTU representative sequences for bacteria were compared against the Silva138 (Pruesse et al., 2007) database using the BLAST (Ye et al., 2006) algorithm with an *e*-value threshold set to 1 × 10^−5^ to obtain the corresponding taxonomic information for each OTU. For fungi, the OTU representative sequences were compared against the Unite 8.2 database (Abarenkov et al., 2010) using the BLAST algorithm with an e-value threshold set to 1 × 10^−5^ to obtain the taxonomic information of the corresponding species.

##### 2.3.3.2 α- and β-diversity analysis

To investigate potential differences in microbiota diversity between different parts of *C. salsa* and *K. foliatum*, we calculated α-diversity using QIIME (v1.8.0), including richness (Chao1), diversity (Simpson), and evenness (Pielou). The resulting α-diversity index data were log-transformed to meet assumptions of normality and homogeneity of variance before being plotted using R (v3.6.0) (Team, R.C, 2014). To visualize compositional differences between communities, we performed Principal Compound Analysis (PCA) using the OTUs dataset, which reflects differences between multiple data sets on a two-dimensional coordinate plot, with axes chosen to maximize the two eigenvalues of the variance values. In the PCA plot, samples that are more similar in composition are closer together. To maximize differences between different parts of *C. salsa*, we further analyzed the metabolic data using Principal Co-ordinates Analysis (PCoA), a method used to visualize the similarities or differences between samples in a dataset, it is based on the distance matrix (other than Euclidean distance) to find principal coordinates.

##### 2.3.3.3 Differential analysis

The study used LDA Effect Size (LEfSe) (Segata et al., 2011) analysis to identify species that differed significantly in abundance between and within groups of *C. salsa* and *K. foliatum*, with the aim of understanding the variation patterns of plant microorganisms in the host rhizosphere-host plant-holoparasite pathway in parasitic plant systems. The Python (v2.7) was utilized for the analysis, employing the ANOVA test to detect species with significant differences in abundance between different subgroups in multiple samples. The threshold value was set at 0.05. Next, the Wilcoxon rank sum test was used to analyze between-group differences among species with significant differences, again with a threshold of 0.05. Finally, the data were downscaled using linear discriminant analysis (LDA), and the influence of species with significant differences was calculated, i.e., the LDA score. The threshold was set at 3.

To evaluate the biological functions of bacterial communities across distinct plant parts, OTUs derived from 16S rRNA sequences were directly imported into PICRUSt (Langille et al., 2013). Subsequently, the KEGG database was employed to forecast the functional gene repertoire of these microbial communities based on the 16S rRNA gene sequences retrieved from the Greengenes database. The analysis aimed to discern significant disparities among the predicted functions, thus, the results obtained from the KEGG database were subjected to further scrutiny using the Kruskal–Wallis test.

##### 2.3.3.4 Network analysis

To investigate the differences in the microbial community structure of *C. salsa*, *K. foliatum*, and their rhizospheres, the study used the molecular ecological network analyses pipeline (MENAP) (Deng et al., 2012) using the online ecological network analysis platform iNap^1^ (Feng et al., 2022). The SparCC program (Friedman and Alm, 2012) was utilized to construct bacterial and fungal co-association networks for CSF, CSL, CSS, H, KFF, KFS, KFR, KFRS, and KFRS. Default parameters were used, and pseudo-*p* values were calculated from 1,000 bootstrap datasets. The obtained associations between operational taxonomic units (OTUs) were filtered based on absolute correlation values >0.6 and pseudo-*p* values <0.05 (Friedman and Alm, 2012). The resulting networks were visualized using Gephi v.0.10.1 (Bastian et al., 2009). Next, the MEN Analysis function in MENAP was used to perform global network attribute analysis, individual node centrality analysis, and module separation analysis. In the module analysis, the Greedy modularity optimization (Newman, 2004) was used to calculate the among-module connectivity (Pi) and within-module connectivity (Zi). Then, all OTUs were assigned to one of four possible network roles: module hubs, connectors, network hubs, and peripherals (Olesen et al., 2006).

##### 2.3.3.5 Procrustes tests

To determine whether the structure of the microbiota of each part of *C. salsa* tracks that of its host plant, *K. foliatum*, and the corresponding inter-root soil microbiota, Procrustes analysis (Peres-Neto and Jackson, 2001) was performed using the R package “vegan” (Dixon, 2003). Based on the first two principal axes of the weighted UniFrac distances (PCoA 1 and PCoA 2), a set of Procrustes analyses was used to calculate the match scores between the corresponding samples. The congruence between two bacterial or fungal communities is indicated by the Procrustes correlation class statistic (*t*_0_), with t_0_ values ranging from 0 to 1, implying complete disagreement and complete agreement, respectively. To investigate which individual bacterial and fungal taxa might contribute to the agreement or disagreement between different parts of *C. salsa* and *K. foliatum* root microbiota, a leave-one-out method analysis was performed in four steps (Miao et al., 2023a). First, all microorganisms belonging to the same phylum-level classification were removed from the data set of a set of samples. Second, the weighted UniFrac distance between the two communities was recalculated. Third, sample scores were obtained from a new PCoA and *t*_0_ (*t*_0_′) was recalculated. Finally, Δ*t* = (*t*_0_′ − *t*_0_) was calculated to assess the importance of excluding a particular bacterial and fungal phylum on the consistency between the two communities.

### 2.4 Correlation analysis of endophytes and differentially enriched metabolites

Intergroup correlation analysis was carried out to identify differentially enriched metabolites in different parts of *C. salsa* associated with endophytes. The ChiPlot online platform^2^ was utilized for the analysis, using Spearman’s correlation coefficient combined with the *Z*-test. The obtained data were visualized as Heatmap plots, illustrating the relationship between the different species and metabolic components in the samples and assessing the correlation between microorganisms and the metabolomic. In the matrix, correlation coefficients were color-coded as orange and green to indicate positive and negative correlations, respectively. The significance of the results was denoted by “*” and “**” for *p* values less than 0.05 and 0.01, respectively (Cui et al., 2018).

## 3 Results and discussion

### 3.1 *C. Salsa* flowers have the most unique metabolic characteristics

**Figure 2:**
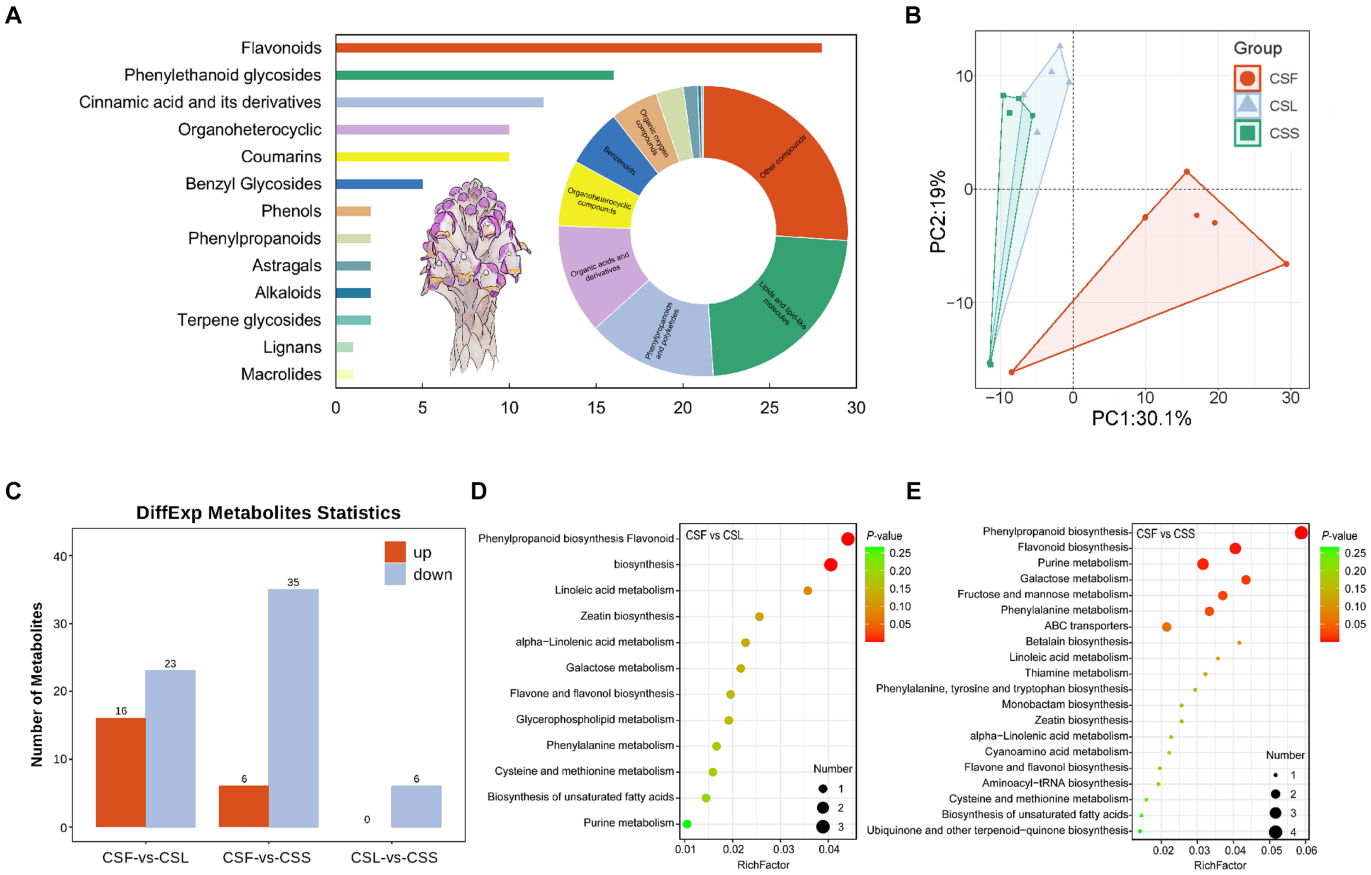
**(A)** Classification of the overall metabolic composition of *C. salsa*. Bar graph is secondary metabolites, ring pie graph is whole metabolites; **(B)** PCA analysis of metabolites of different parts of *C. salsa*; **(C)** Histogram of differentially enriched metabolites statistics among different parts of *C. salsa*; **(D,E)** KEGG enrichment analysis of differentially enriched metabolites among different parts of *C. salsa*. CSF, flower of *C. salsa*; CSL, leaf of *C. salsa*; CSS, stem of *C. salsa*; each analysis comprises six biological replicates.

The qualitative analysis of mass spectrometry information was conducted using both public databases and a proprietary database, resulting in information on 491 compounds (Figure 2A and Supplementary Table S1). Comprising mainly lipids and lipid-like compounds, phenylpropanoids and polyketides, as well as organic acids and derivatives. These compounds were categorized into primary and secondary metabolites, consisting of 398 primary metabolites and 93 secondary metabolites. Secondary metabolites were primarily composed of flavonoids, phenylethanol glycosides, and cinnamic acid and its derivatives. Analysis of metabolite composition in *C. salsa* flowers, scale leaves, and fleshy stems indicated that flowers contained the most metabolites (479), followed by scale leaves (469), and fleshy stems (454). Among these three parts of *C. salsa*, there were 442 common metabolites, while the flower and scale leaf contained 21 and 1 unique metabolites, respectively, and the fleshy stem had no unique metabolites (Supplementary Figure S1). PCA analysis revealed clear separation of samples from flower parts, while samples from scaly leaf and fleshy stem overlapped, suggesting relatively similar metabolite composition and content in the scaly leaf and fleshy stem, and significant differences in the flower (Figure 2B).

As shown in Figure 2C and Supplementary Table S2, the OPLS-DA-based metabolite differential analysis revealed that there were 39 differentially enriched metabolites in scale leaves compared with flowers. Likewise, 41 differentially enriched metabolites were identified in fleshy stems compared with flowers. Six differentially enriched metabolites were found in fleshy stems compared with scale leaves. To investigate the biological significance of the differentially enriched metabolites in different parts of *C. salsa*, metabolic pathway enrichment analysis was performed using the KEGG. As depicted in Figures 2D,E, a total of 12 pathways were enriched in the CSF-CSL group. The most significant pathways enriched were phenylpropanoid biosynthesis and flavonoid biosynthesis. In the CSF-CSS group, 25 pathways were enriched. The most significantly enriched pathways enriched in this group were phenylpropanoid biosynthesis, flavonoid biosynthesis, and purine metabolism. No metabolic pathways were enriched in the CSL-CSS group.

Previous investigations on *C. salsa* have primarily emphasized substances such as phenylethanol glycosides, with no examination of the flavonoid composition in this species (Lei et al., 2020). Moreover, the flowers of *C. salsa* exhibited the most distinctive metabolic profile, with 21 unique metabolites, including lipids and lipid-like molecules, organic acids and derivatives, and flavonoids. Some of these metabolites have been shown to possess significant pharmacological effects, for instance, (2S)-liquiritigenin, which has inhibitory effects on a variety of cancer invasion and metastasis (Tang et al., 2022), (S)-pinocembrin, which has anti-inflammatory and antixodant effects and improves endothelial cell function, protects against neurological damage, and anti-tumor effects (Gong, 2021), and dihydroquercetin, which has anti-inflammatory and antioxidant properties, modulates enzyme activity, and affects lymphocyte behavior (Dong et al., 2020). Compared to the flowers, the metabolic characteristics of the leaves do not differ significantly from the stems, and the leaves have only one unique metabolite, which may be due to the degeneration of the leaves of *C. salsa* due to parasitism.

### 3.2 The diversity of microbial community assembly characteristics

**Figure 3:**
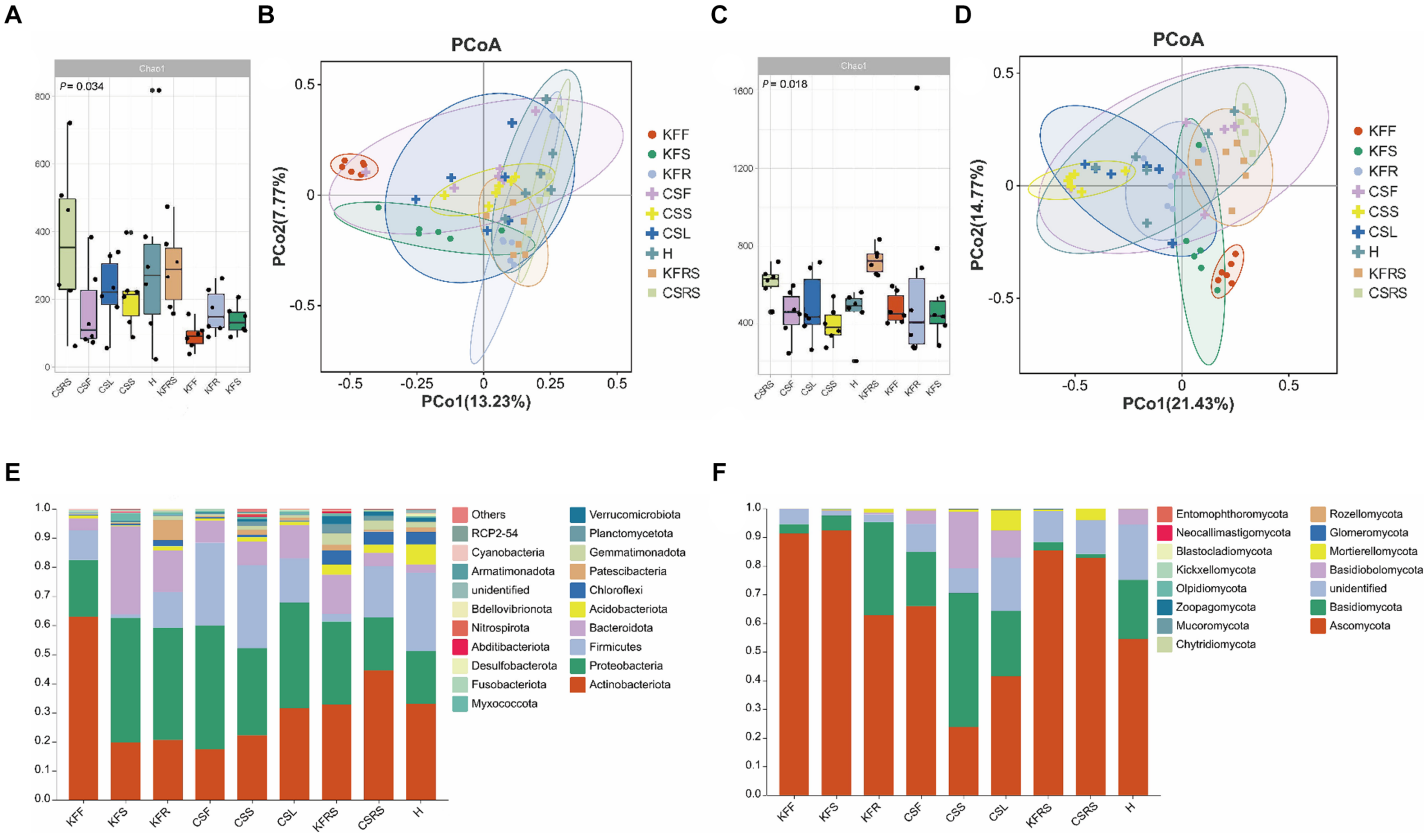
Analysis of microbial diversity. **(A)** Alpha diversity analysis of bacteria; **(B)** PCoA analysis of bacteria; **(C)** alpha diversity analysis of fungi; **(D)** PCoA analysis of fungi; **(E)** analysis of species composition of bacteria. **(F)** Analysis of species composition of fungi. KFF, flower of *K. foliatum*; KFS, stem of *K. foliatum*; KFR, root of *K. foliatum*; KFRS, rhizosphere of *K. foliatum*; CSF, flower of *C. salsa*; CSL, leaf of *C. salsa*; CSS, stem of *C. salsa*; H, haustorium of *C. salsa*; KFRS, rhizosphere of *C. salsa*; each analysis comprises six biological replicates.

In terms of bacterial diversity (Figures 3; Supplementary Figure S2), flowers of both *K. foliatum* and *C. salsa* exhibited the lowest α diversity, while fleshy stems and haustoria of *C. salsa* exhibited higher bacterial abundance compared to the roots of *K. foliatum*, albeit lower in terms of diversity and evenness. The rhizosphere of both plants showed the highest α diversity. β-diversity analysis revealed significant differences between groups in different parts of *K. foliatum*, while no significant differences were observed in different parts of *C. salsa*. The top four bacterial phyla with the highest relative abundance in all samples were Actinobacteriota, Proteobacteria, Firmicutes, and Bacteroidota, respectively. Bacteria unique to different parts of *C. salsa* accounted for 76.45% of the total bacteria, while bacteria unique to different parts of *K. foliatum* accounted for 78.85%. The proportion of shared bacteria between *C. salsa* and *K. foliatum* accounted for 33.51 and 47.88% of their respective total bacterial species (Supplementary Figure S3). In terms of fungi (Figures 3; Supplementary Figure S2), the abundance of fungal communities was similar in all parts of *K. foliatum*, while the roots exhibited the lowest diversity and evenness. On the other hand, the haustorium of *C. salsa* had the highest fungal abundance, and the fleshy stems had the lowest fungal diversity and evenness. The rhizosphere of *K. foliatum* exhibited the highest α-diversity of fungi, while the diversity and evenness of fungi of the rhizosphere of *C. salsa* was lower than that of the haustorium. β-diversity analysis indicated no significant differences in endophytic fungi between *K. foliatum* and *C. salsa*. The top three fungal phyla with the highest relative abundance among all samples were Ascomycota, Basidiomycota, and Basidiobolomycota, respectively. Moreover, 53.74% of the total fungi were unique to different parts of *C. salsa*, 54.10% of the total fungi were unique to different parts of *K. foliatum*, and 54.10% of the total fungi were unique to different parts of *C. salsa* and *K. foliatum*. *C. salsa* and *K. foliatum* accounted for 60.48 and 67.83% of their respective total fungi species (Supplementary Figure S3).

**Figure 4:**
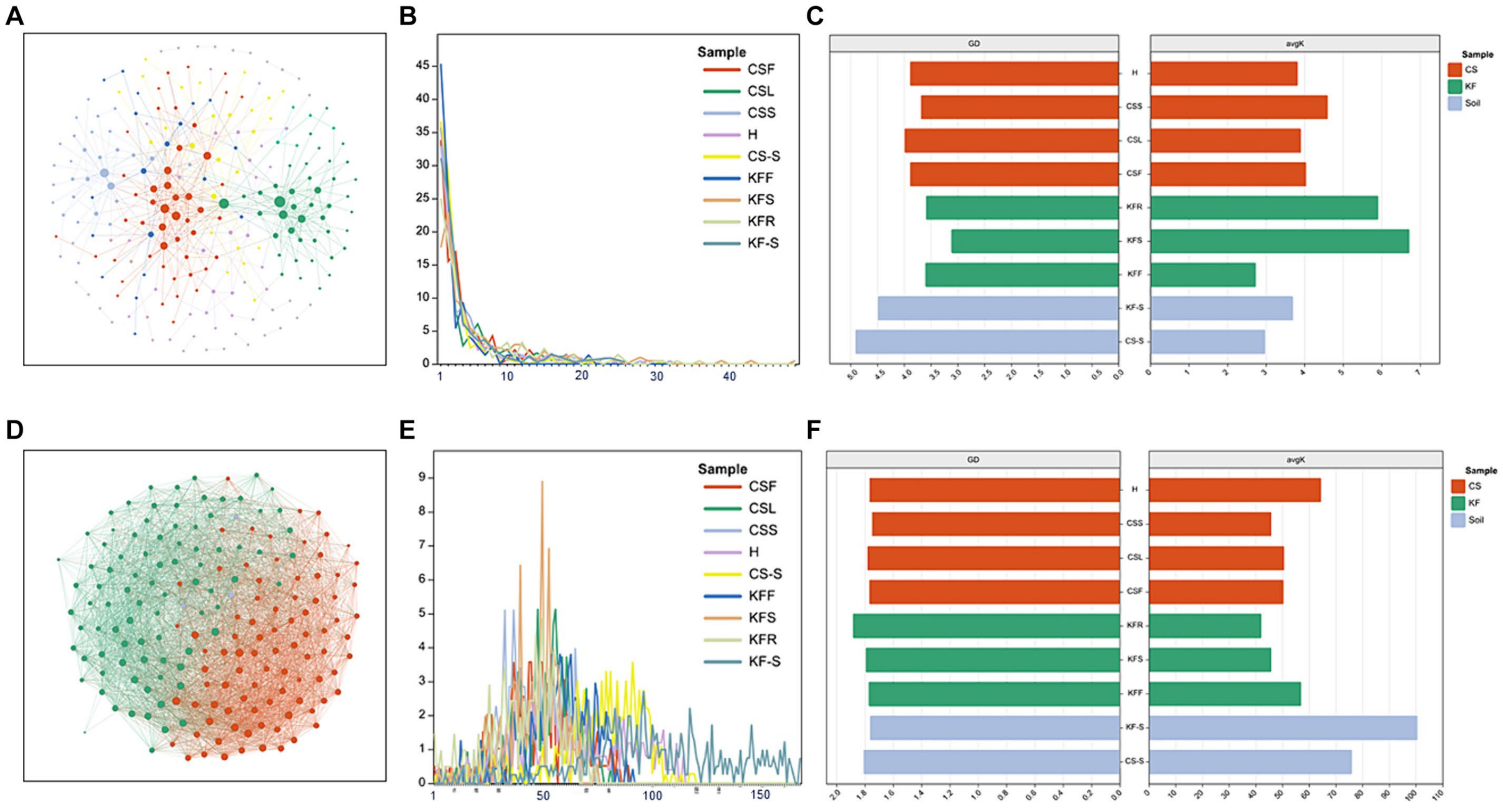
Network characterization of microorganisms. **(A,D)** Network analysis of bacteria and fungi of fleshy stems of *C. salsa* (CSS); **(B,E)** degree distribution (number of associations per node) among community types of bacteria and fungi network; **(C,F)** average path distance (GD) and average degree (avgK) of bacteria and fungi network.

The network characterization revealed significant variations in the structure of the microbial communities between the two species. Figures 4 and Supplementary Figure S4 show that the endophytic bacterial networks in the two rhizosphere groups were more similar than in the plants. The average degree (avgK) and the degree distribution of bacteria were lower in *C. salsa* than its host *K. foliatum*. Despite this, the average path distance (GD) of *C. salsa* was similar to *K. foliatum*. *C. salsa* had a simpler endophytic bacterial network with fewer co-associated OTUs, but a higher number of modules than *K. foliatum*. Furthermore, both plants showed reduced association between bacterial community members in floral parts compared to belowground parts. The modularity analysis (Supplementary Figure S6) of the endophytic bacterial network shows that *K. foliatum* had more module hubs, network hubs and connectors than *C. salsa*, indicating that *K. foliatum* had higher intra-module and inter-module connectivity. Network hubs and connectors mainly belonged to Actinobacteriota, Firmicutes, Bacteroidota and Proteobacteria, which indicated that these bacteria played an important role in the endophytic bacterial network of the whole parasitic plant system. As demonstrated in Figures 4 and Supplementary Figure S5, the fungal networks of the two rhizosphere groups exhibited more similar structures. The average degree, average path distance, and degree distribution of the fungal networks of *C. salsa* and *K. foliatum* were essentially alike and significantly higher than those of the bacterial networks. This finding suggests that the endophytic fungal networks of *C. salsa* and *K. foliatum* were more intricate than the endophytic bacterial networks. Notably, all fungal networks were internally divided into three modules. However, in the fleshy stems and haustorium of *C. salsa*, OTUs were unevenly distributed among the three modules. Most OTUs were mainly distributed in two modules, indicating that the fungal community members of haustorium and fleshy stems of *C. salsa* were more closely associated with each other than with scale leaves and flowers. The modularity analysis of the endophytic fungal network (Supplementary Figure S7) revealed that the scale leaf of *C. salsa* had one module hub, belonging to Ascomycota, and all fungal OTUs in the fleshy stems were peripherals. In contrast, in other parts of *C. salsa* and all parts of *K. foliatum*, there were no module hubs and network hubs. In different parts of *C. salsa*, the connectors of the endophytic fungal network belonged mainly to Ascomycota and Basidiomycota, while in different parts of *K. foliatum*, the connectors of the endophytic fungal network were predominantly Ascomycota. In the rhizosphere, the connectors of the endophytic fungal network were mainly Ascomycota and Basidiomycota.

Our findings reveal a gradual decrease in bacterial diversity from the host to the parasitic plant, which aligns with the reduction in morphological and anatomical structures of the parasitic plant (Tate, 1925) and enriches the previous conclusions on “parasitic reduction syndrome” (Colwell, 1996). Furthermore, the endophytic bacteria residing in the root tissues of *C. salsa* and its host plant *K. foliatum* exhibit a remarkable difference in their composition compared to other plant parts. Previous studies have shown that changes in the functional characteristics and ecological strategies of plants can lead to differences in endophytic bacterial communities at different sites among plant species (Fitzpatrick et al., 2018), and our findings are consistent with this paradigm of bacterial diversity and composition in the roots. In addition, bacterial populations were more alike among distinct regions of *C. salsa* in contrast to *K. foliatum*, and the intricate nature of bacterial networks was lesser than that of *K. foliatum*. This finding reinforces the idea that bacterial-bacterial interactions and ecological niche-based mechanisms have a limited contribution towards the establishment of endophytic bacterial communities in parasitic plants. One potential rationale could explain this phenomenon: the organs of distinct parts of parasitic plants have a higher uniformity in function compared to autotrophs, resulting in a greater overlap of bacterial community habitats among different regions of parasitic plants. Nonetheless, the endophytic fungi of *C. salsa* and *K. foliatum* do not exhibit these features, and the fungi reveal a comparable community structure across distinct regions of both host and parasitic plants, and the complexity of the network is also alike. This suggests that plants display reduced selectivity in recruiting fungi, likely due to the direct colonization of aerial plant parts by airborne fungal spores, in addition to root colonization by fungi (Lucas et al., 1992; Nageen et al., 2021). Other potential factors contributing to this phenomenon, such as plant growth cycles, ecological niche differences, or host plant species, require more in-depth study in the future. Simultaneously, the outcomes indicate that rhizosphere microorganisms have a greater diversity and more complex taxonomic groups and microbial networks than the endophytic microorganisms of *K. foliatum* and *C. salsa*. The rhizosphere serves as a conduit for nutrient uptake by plants and acts as the first line of defense against various abiotic and biotic stresses. Plants alter the rhizosphere milieu by absorbing water, salts, mineral elements, oxygen, and nutrients from the rhizosphere, creating deposits, and producing exudates (Shi et al., 2016; Freilich et al., 2018; Bulgarelli et al., 2022). Hence, the exceptional environment of the rhizosphere contributes to the diversity of its microbial communities.

### 3.3 Endophytes exhibit a preference for micro niches

**Figure 5:**
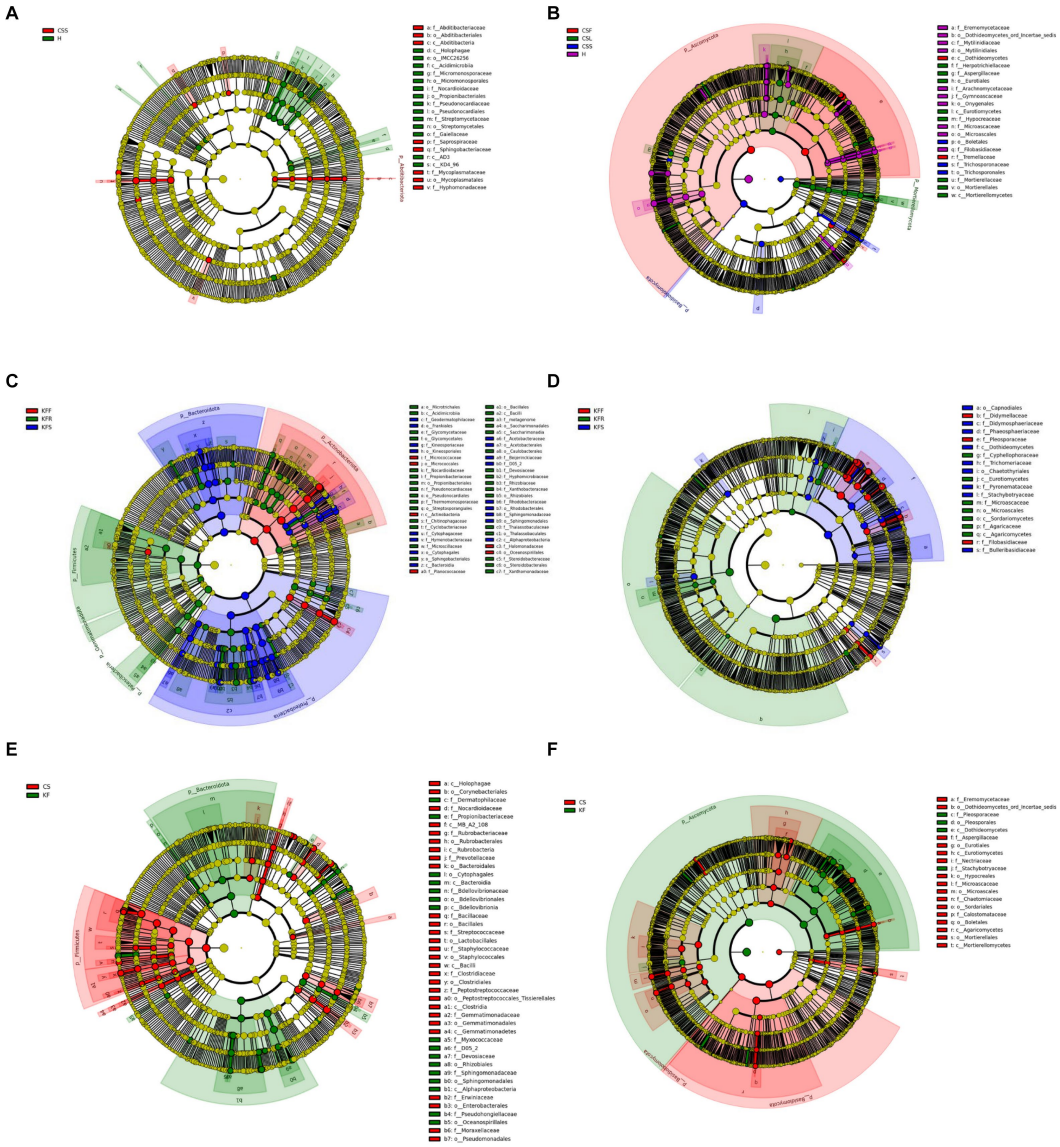
Evolutionary branching diagram of LEfSe analysis of endophytic microorganisms based on taxonomic information. **(A,B)** Bacteria **(A)** and fungi **(B)** in different parts of *C. salsa*. **(C,D)** Bacteria **(C)** and fungi **(D)** in different parts of *K. foliatum*. **(E,F)** Bacteria **(E)** and fungi **(F)** in *C. salsa* and *K. foliatum*. This diagram uses circles radiating from the center to represent classification levels from phylum to genus (or species). Each small circle represents a classification at a different level, and the diameter of the circle is proportional to the relative abundance. Species with no significant differences are colored yellow, while biomarker-following groups are colored accordingly. In the diagram, red nodes represent microbial communities that play an important role in the red group, while green nodes represent microbial communities that play an important role in the green group. Other colored circles have the same meaning. The English letters in the diagram correspond to the species names in the legend on the right.

In terms of endophytic bacteria and fungi distribution in different parts of *C. salsa* and *K. foliatum*, several patterns emerged (Figure 5). In *C. salsa*, the bacterial order Streptomycetales exhibited the highest relative abundance in the haustorium. As for fungi, the order Dothideomycetes within the phylum Ascomycota was most prevalent in the flowers, Eurotiales of Ascomycota and Mortierellales of Mortierellomycota were most abundant in the scale leaf. Additionally, in the haustorium, Microascales, Onygenales, Mytilinidiales, and Eremomycetaceae of Ascomycota displayed the highest relative abundance. In contrast, in *K. foliatum*, Actinobacteriota had the highest relative abundance in flowers, particularly with Micrococcaceae playing a decisive role. The stem was primarily colonized by Bacteroidota and Proteobacteria, while Firmicutes, Gemmatimonadota, and Patescibacteria were the most abundant in roots. Among fungi, Agaricomycetes, Sordariomycetes, and Eurotiomycetes were the most abundant in roots. Additionally, Dothideomycetes Capnodiales, Didymosphaeriaceae, and Phaeosphaeriaceae were the most abundant in stems, while Pleosporaceae and Didymellaceae of Dothideomycetes were most abundant in flowers. Regarding the comparison of the endophytic microbial community between *C. salsa* and *K. foliatum*, Firmicutes were more abundant in *C. salsa*, while Alphaproteobacteria of Proteobacteria and Bacteroidia of Bacteroidota were more abundant in *K. foliatum*. Among fungi, *K. foliatum* had higher levels of Dothideomycetes of Ascomycota compared to *C. salsa*, whereas Eurotiomycetes, Hypocreales, Microascales, Sordariales of Ascomycota, and Basidiomycota were more abundant in *K. foliatum*. In contrast, Boletales were more abundant in *C. salsa*.

The PICRUSt analysis revealed a total of 18 differentially enriched pathways (Supplementary Figure S8). Within the realm of Metabolism, pathways encompassing amino acid metabolism, carbohydrate metabolism, and lipid metabolism were identified. Furthermore, pathways associated with cellular processes such as cell growth and death, cell motility, and cellular community prokaryotes were observed. Additionally, environmental information processing pathways including membrane transport and signal transduction were detected, along with genetic information processing pathways like replication and repair, and transcription. Notably, the top three most abundant pathways across all plant parts were carbohydrate metabolism, amino acid metabolism, and metabolism of cofactors and vitamins. Each of these pathways exhibited its highest abundance in the flowers of the host plant. Unlike the host plant, where the pathway for amino acid metabolism showed a slightly higher abundance in the haustorium compared to the flowers, the remaining two pathways displayed their highest abundance in the flowers.

Differential abundance analyses of endophytes showed the presence of microorganisms with significant abundance differences between the parasitic and host plants and between their respective different parts. Our suggestion is that the disparity in the abundance of endophytes between host and parasitic plants is consistent with the concept of species specificity and selective specificity between endophytes and hosts, as previously proposed (Rai and Agarkar, 2016). Only the microorganisms capable of adapting to the internal environment of the plant are likely to survive and reproduce (Huang et al., 2017). Conversely, the surviving microorganisms have a beneficial impact on the growth of their host plant. This phenomenon may be the outcome of co-evolution and natural selection between the microorganisms and their host plant (Ambrose et al., 2014; Zeng et al., 2023).

### 3.4 Specific microbial taxa influence the consistency of microbial communities between parasitic plants and their hosts

**Figure 6:**
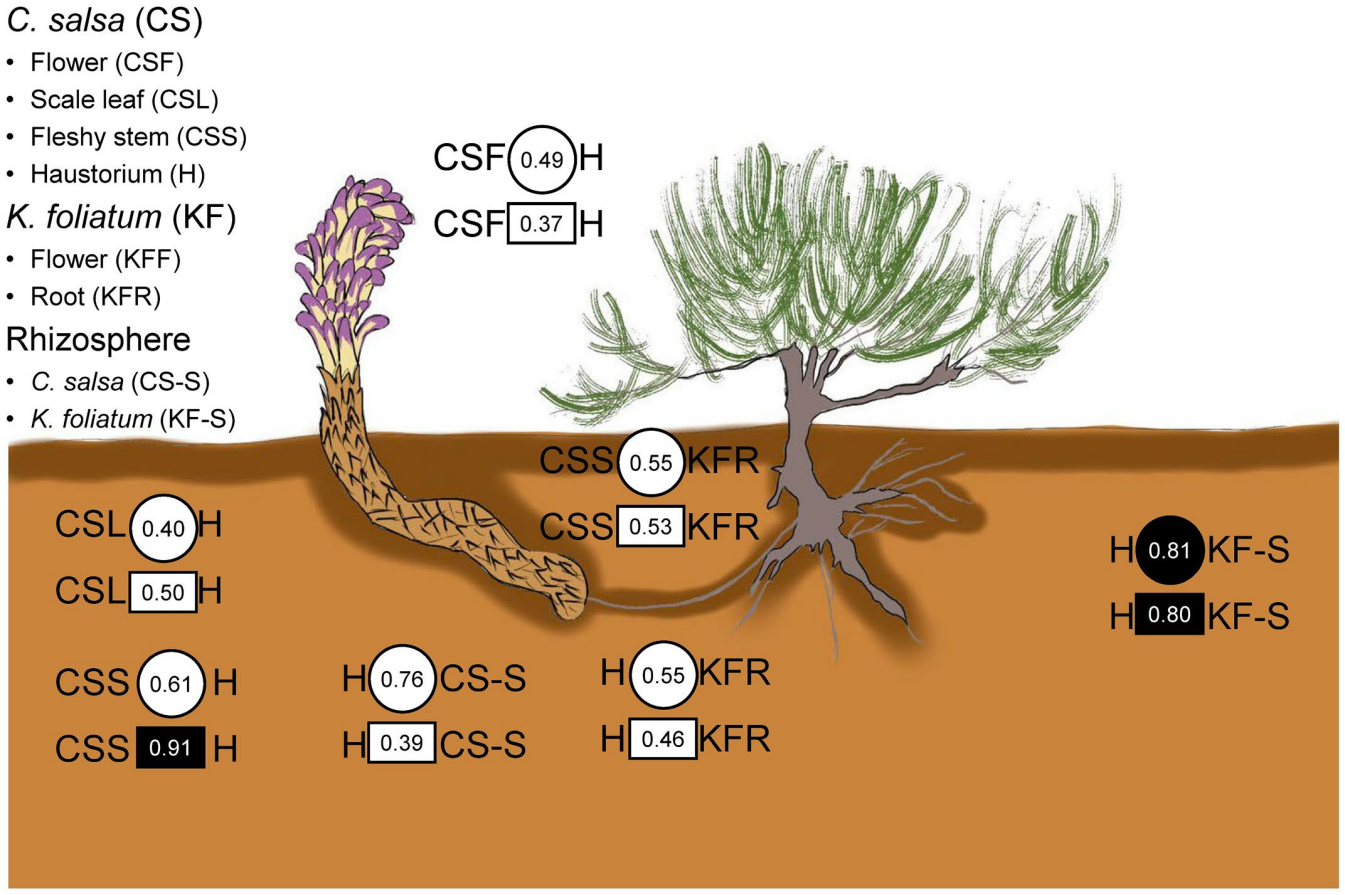
Congruence in composition changes of corresponding plant parts and the rhizosphere of *C. salsa* and *K. foliatum*. Circles and squares indicate Procrustes correlations for endophytic bacteria and fungi, respectively. Filled shapes indicate *p* < 0.05.

The results of the procrustes analysis (Figure 6 and Supplementary Table S3) revealed that microbial communities in *C. salsa* differed significantly between flowers and scale leaves compared to the haustorium (bacteria: CSF vs. H *t*_0_ = 0.49, *p* = 0.58, CSL vs. H *t*_0_ = 0.40, *p* = 0.73; fungi: CSF vs. H *t*_0_ = 0.37, *p* = 0.57, CSL vs. H *t*_0_ = 0.50, *p* = 0.38), however, the microbial community in the fleshy stems was consistent with that of the haustorium, (bacteria: CSS vs. H *t*_0_ = 0.61, *p* = 0.30; fungi: CSS vs. H *t*_0_ = 0.91, *p* = 0.01). Within *K. foliatum*, the endophytic bacterial microbial communities in both flowers and stems of *K. foliatum* were dissimilar to those of the roots, (KFF vs. KFR *t*_0_ = 0.38, *p* = 0.88, KFS vs. KFR *t*_0_ = 0.47, *p* = 0.44), while in the fungal microbial communities, flowers were dissimilar to the roots, but stems were similar to the roots, (KFF vs. KFR *t*_0_ = 0.41, *p* = 0.58, KFS vs. KFR *t*_0_ = 0.69, *p* = 0.13). The microbial communities present in the fleshy stems and haustorium of *C. salsa* were found to be similar to those present in the roots of *K. foliatum*, however, the observed similarity was not significant (bacteria: CSS vs. KFR *t*_0_ = 0.55, *p* = 0.30, H vs. KFR *t*_0_ = 0.55, *p* = 0.20; fungi: CSS vs. KFR *t*_0_ = 0.53, *p* = 0.44, H vs. KFR *t*_0_ = 0.46, *p* = 0.64). Of particular interest is that the endophytic microbial community of the haustorium displayed a notably high and significant agreement with the rhizosphere of *K. foliatum* (bacteria: *t*_0_ = 0.81, *p* = 0.05; fungi: *t*_0_ = 0.80, *p* = 0.03).

The leave-one-out analysis revealed that several bacterial and fungal phyla were closely associated with the concordance or inconsistency between the microbial communities of *C. salsa* and *K. foliatum*. As shown in Supplementary Figure S9, in terms of bacteria, the consistency of bacterial communities of fleshy stems of *C. salsa* with the roots of *K. foliatum* was significantly reduced after excluding Patescibacteria (Δ*t* = −0.39), Actinobacteriota (Δ*t* = −0.38), Acidobacteriota (Δ*t* = −0.39), and Nitrospirota (Δ*t* = −0.39). The consistency of bacterial communities in the roots of *K. foliatum* with the scale leaves, fleshy stems, and haustorium of *C. salsa* was reduced after the exclusion of Firmicutes. Given that Ascomycota and Basidiomycota constitute the primary fungal phyla in roots, analyzing them at the fungal phylum level lacks meaningful insight. Consequently, we conducted a “leave-one-out” analysis at the fungal class level (Supplementary Figure S10). The results revealed that excluding each fungal class enhanced the consistency of fungal communities in the scale leaves of *C. salsa* and the roots of *K. foliatum*, with the most notable changes in consistency observed upon excluding Chytridiomycetes (Δ*t* = 0.34), Glomeromycetes (Δ*t* = 0.38), and Rhizophlyctidomycetes (Δ*t* = 0.37). While the exclusion of most fungal classes decreased the consistency of fungal communities in the flowers of *C. salsa* with the roots of *K. foliatum*, the most substantial change occurred with the exclusion of Rhizophlyctidomycete (Δ*t* = −0.31). Furthermore, the fungal community congruence between the haustorium and the roots of *K. foliatum* significantly increased after excluding Orbiliomycetes (Δ*t* = 0.53).

These findings suggest that a few bacterial and fungal phyla are closely linked to the concordance or inconsistency between microbial communities in the two plant species. Furthermore, we observed that different microbial taxa contributed to the concordance of microbial communities in various parts of *C. salsa* and in the roots of *K. foliatum*. These observations imply that the characteristics underlying the assembly of microbial communities in different parts of parasitic plants are not identical (Fitzpatrick and Schneider, 2020).

### 3.5 Endophytes and the accumulation of metabolites correlate in various tissues of *C. Salsa*

**Figure 7:**
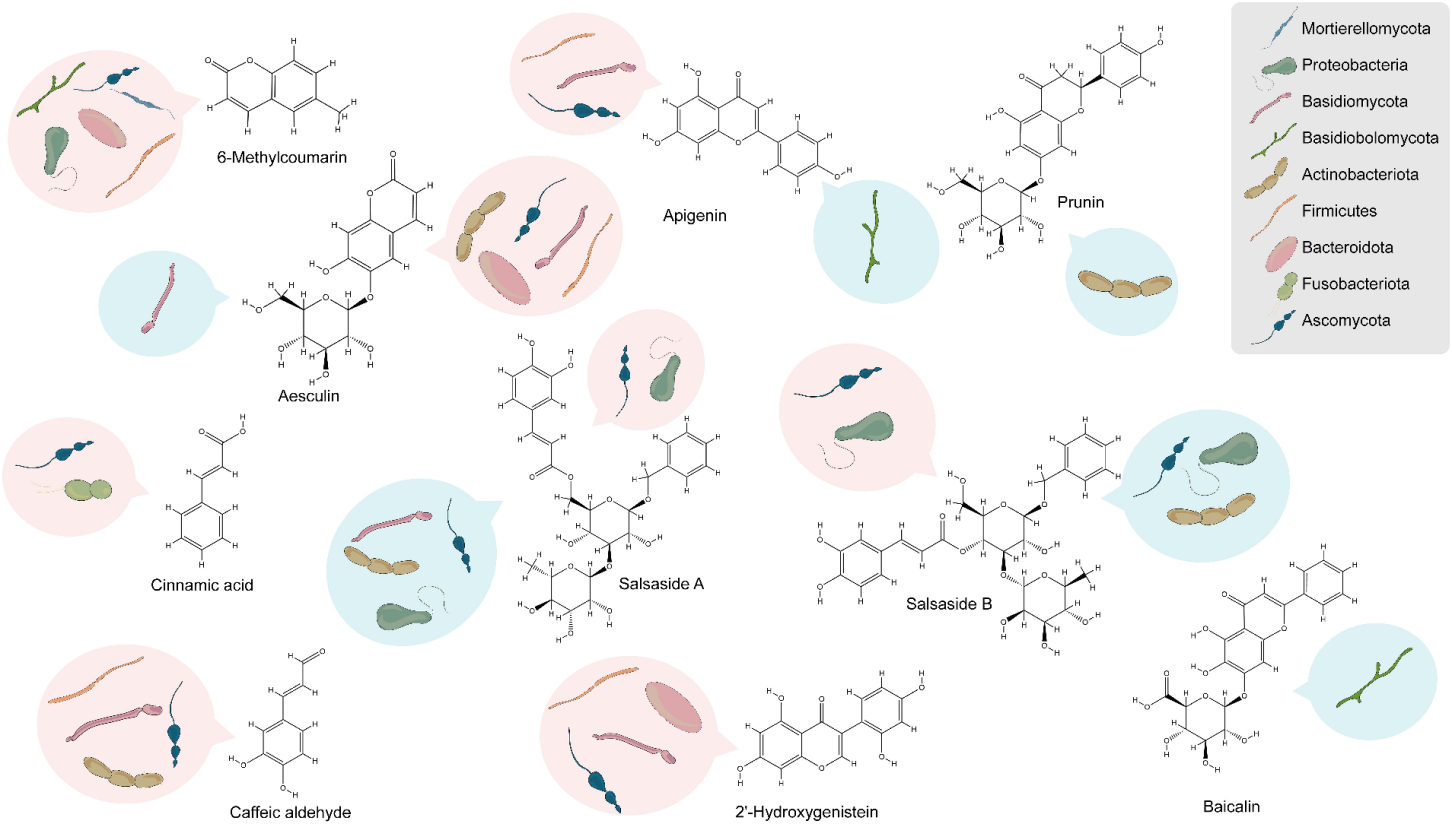
Analysis of endophytes associated with the accumulation of secondary metabolites based on phylum-level classification. Red represents positive correlation, blue represents negative correlation.

The study’s findings (Supplementary Figures S11–S16) reveal that 31, 41, and 4 of the differentially enriched metabolites in groups CSF vs. CSL, CSF vs. CSS, and CSL vs. CSS respectively, were significantly associated with endophytic bacteria, and these endophytic bacteria belonged mainly to Bacteroidota, Proteobacteria, Actinobacteriota, and Firmicutes. Additionally, 32, 46, and 5 differentially enriched metabolites were significantly associated with endophytic fungi, and these endophytic fungi belonged mainly to Ascomycota and Basidiomycota. Overall, among all differentially enriched metabolites significantly associated with endophytes, 10 were secondary metabolites. These include 6-methylcoumarin, aesculin, 2′-hydroxygenistein, caffeic aldehyde, salsaside A, salsaside B, apigenin, prunin, cinnamic acid, and baicalin (Figure 7 and Supplementary Table S4).

Nine differentially enriched secondary metabolites were significantly associated with endophytes in CSL compared to CSF, with only 6-methylcoumarin and aesculin showing higher enrichment in CSL than in CSF. Similarly, nine differentially enriched secondary metabolites were significantly associated with endophytes in CSS compared to CSF, and only 6-methylcoumarin exhibited greater enrichment in CSS than in CSF. To rephrase, 6-methylcoumarin demonstrated a deficiency in CSF, potentially linked to the deficiency of bacteria such as *Hymenobacter*, *Rufibacter*, *Rubellimicrobium*, *Altererythrobacter*, *Paracoccus*, and *Mycoplasma*, as well as fungi belonging to *Alternaria* and *Dematiopleospora*. Furthermore, secondary compounds significantly enriched in CSF, namely apigenin, caffeic aldehyde, and 2′-hydroxygenistein, exhibited significant positive correlations with the abundance of bacteria in the genus *Planomicrobium*.

Notably, the endophytes that showed significant positive correlation with the compound salsaside, which is specific to *C. salsa*, were *Pantoea*, *Bacillus*, *Planomicrobium*, *Alternaria*, *Neocamarosporium*, *Aureobasidium* and other unidentified genera, and the endophytes that showed significant negative correlation were *Enterobacter*, *Rubrobacter*, *Nocardioides*, *Aspergillus*, *Pseudogymnoascus*, *Dematiopleospora*, *Alternaria* and *Trichosporon*.

Jia et al. (2016) performed a thorough review of published data spanning 30 years regarding the correlation between medicinal plants and endophytes. They concluded that if this relationship is well comprehended, production conditions are optimized, and cultivable endophytes related to medicinal plants are effectively established, medicinal compounds can be produced proficiently at biotechnological scales. Additionally, other researchers have demonstrated the significance of endophytic bacterial diversity in pivotal trophic interactions concerning parasitic plants and their host plants (Zhang et al., 2006; Behie et al., 2012; Ahsan et al., 2024). Our correlation analysis of differentially enriched metabolites in different parts of *C. salsa* with endophytes indicated a significant and positive correlation between most endophytes and differential secondary metabolites. Bacterial taxa such as Bacteroidota, Proteobacteria, Actinobacteriota, Firmicutes, and fungal taxa such as Ascomycota and Basidiomycota played a major role in this correlation. We identified 10 secondary metabolites that showed high association with endophytes, namely 6-methylcoumarin, aesculin, 2′-hydroxygenistein, caffeic aldehyde, salsaside A, salsaside B, apigenin, prunin, cinnamic acid, and baicalin, which were shown to have anticancer, antioxidant, anti-inflammatory, and hypolipidemic effects (Qi et al., 2021; Wu et al., 2022; Lin et al., 2023).

Prior research has established the existence of a mutually beneficial relationship between plants and endophytes, wherein the internal environment of the host plant plays a crucial role in shaping the ecological composition of endophytes (Jia et al., 2016). Endophytes have been shown to facilitate plant development and metabolism through various mechanisms, including the stimulation of host defense responses, alteration of host metabolic patterns, and influence on the accumulation of host compounds (Qawasmeh et al., 2012; Saikkonen et al., 2013; Neyaz et al., 2022; Nischitha and Shivanna, 2024). For example, Cui et al. utilized a combination of metabolomics and high-throughput sequencing techniques to demonstrate the inseparable relationship between endophytes of *Cynomorium songaricum* and metabolites in different production regions (Cui et al., 2019). In this investigation, we present novel evidence indicating a strong correlation between secondary metabolites derived from distinct parts of *C. salsa* and endophytes. Our study’s findings demonstrate that the secondary metabolites present in different parts of *C. salsa* are intricately associated with endophytes. This discovery provides a theoretical foundation to better understand the interactions between *C. salsa* and endophytes, facilitating sustainable production and rational exploitation of medicinal resources derived from *C. salsa*.

## 4 Conclusion

This study systematically investigated the metabolic characteristics of various components of *C. salsa* and their correlation with endophytic bacteria. Furthermore, it explores the similarities and differences in endophytes between *C. salsa* and its host, *K. foliatum*, by employing a combination of plant metabolomics and microbiomics. The findings reveal that the metabolic features of *C. salsa* flowers are particularly distinctive, and the presence of 10 secondary metabolites is significantly associated with Bacteroidota, Proteobacteria, Ascomycota and Basidiomycota in *C. salsa*. Moreover, our investigation revealed distinct microbial community characteristics between endophytic bacteria and endophytic fungi. The outcomes of this study contribute to a comprehensive understanding of microecological investigations on endophytes of *C. salsa* and their interactions with plants. They also shed light on the endophytic microbial characteristics of the entire parasitic plant system, comprising both parasitic plants and their hosts. Furthermore, this study presents novel insights for developing endophyte-based strategies aimed at enhancing the quality of *C. salsa*.

## Data availability statement

The datasets presented in this study can be found in online repositories. The names of the repository/repositories and accession number(s) can be found at: https://www.ncbi.nlm.nih.gov/genbank/, PRJNA976640 https://www.ncbi.nlm.nih.gov/genbank/, PRJNA976507.

## Author contributions

ZF: Supervision, Writing – review & editing, Investigation, Methodology, Writing – original draft. YJM: Methodology, Writing – review & editing. XS: Methodology, Writing – review & editing. YZ: Writing – review & editing. GML: Writing – review & editing. JP: Writing – review & editing. LFH: Writing – review & editing, Supervision.

## Footnotes

^1^http://mem.rcees.ac.cn:8081

^2^https://www.chiplot.online/index.html
