# Supplementary material for: Characterization of microbial community assembly in parasitic plant systems and the influence of microorganisms on metabolite accumulation in parasitic plants: case study of Cistanche salsa and Kalidium foliatum
Source: Front Microbiol. 2024 Jul 25;15:1279536. doi: 10.3389/fmicb.2024.1279536 (PMC11312099; doi:10.3389/fmicb.2024.1279536)
Supplement: Supplementary file 1 [file Data_Sheet_1.PDF]

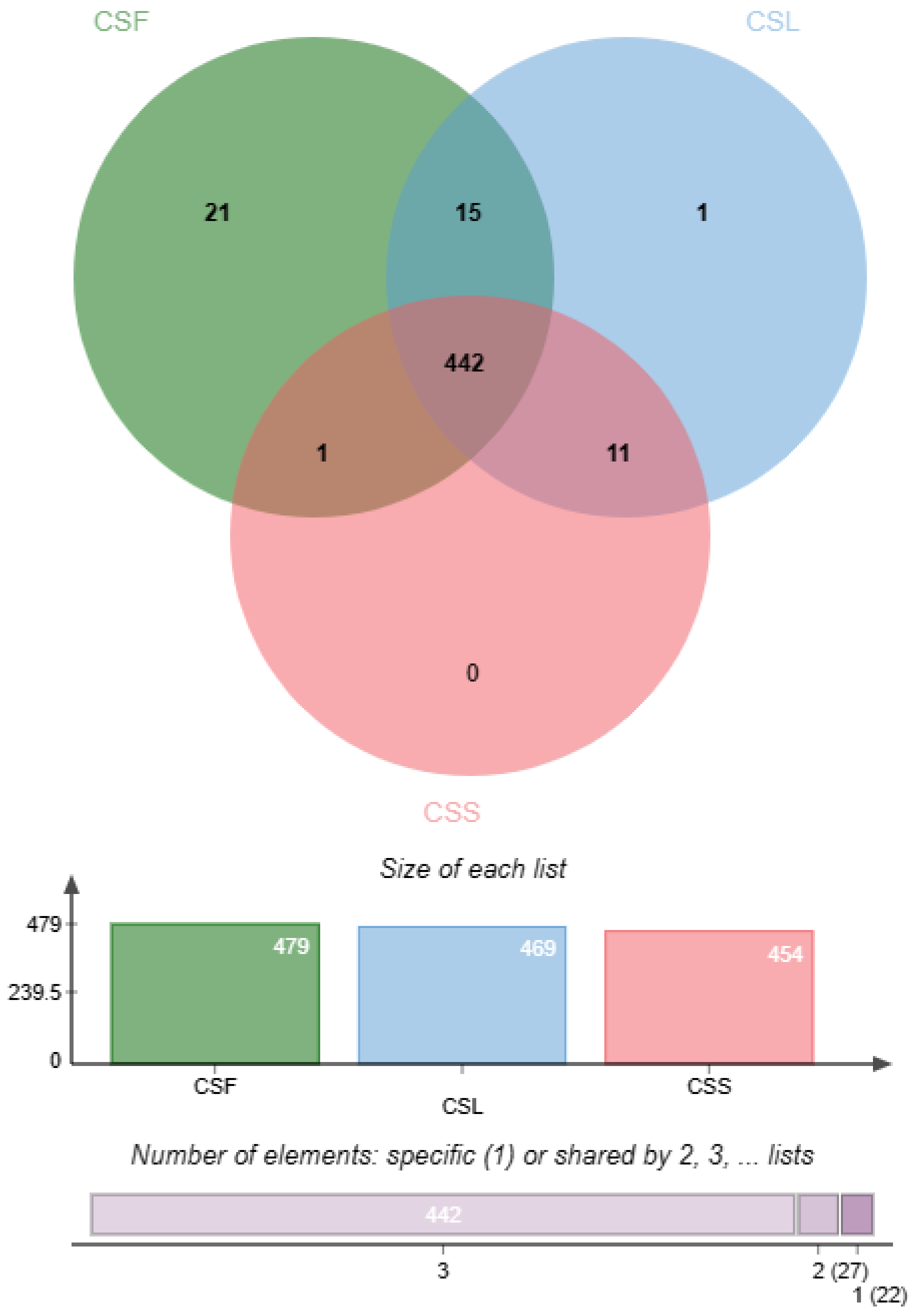

Figure S1. Metabolites common and unique to different parts of *C. salsa*.

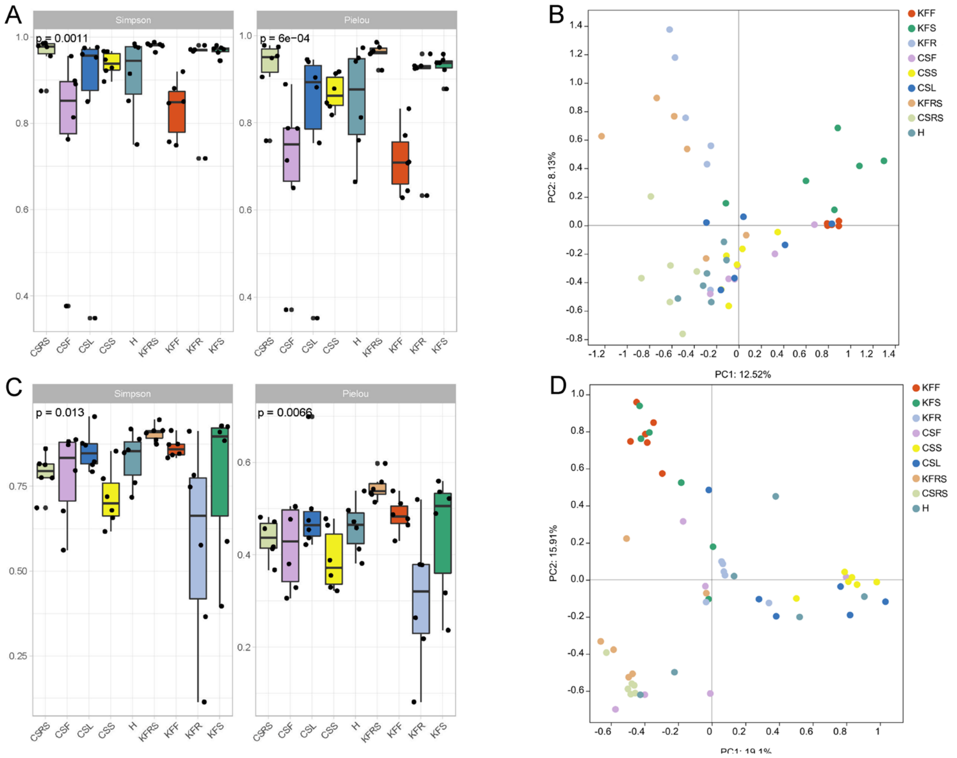

Figure S2. Analysis of the diversity of bacteria and fungi.

Note: A. alpha diversity analysis of bacteria. B. PLS-DA analysis of bacteria. C. alpha diversity analysis of fungi. D. PLS-DA analysis of fungi.

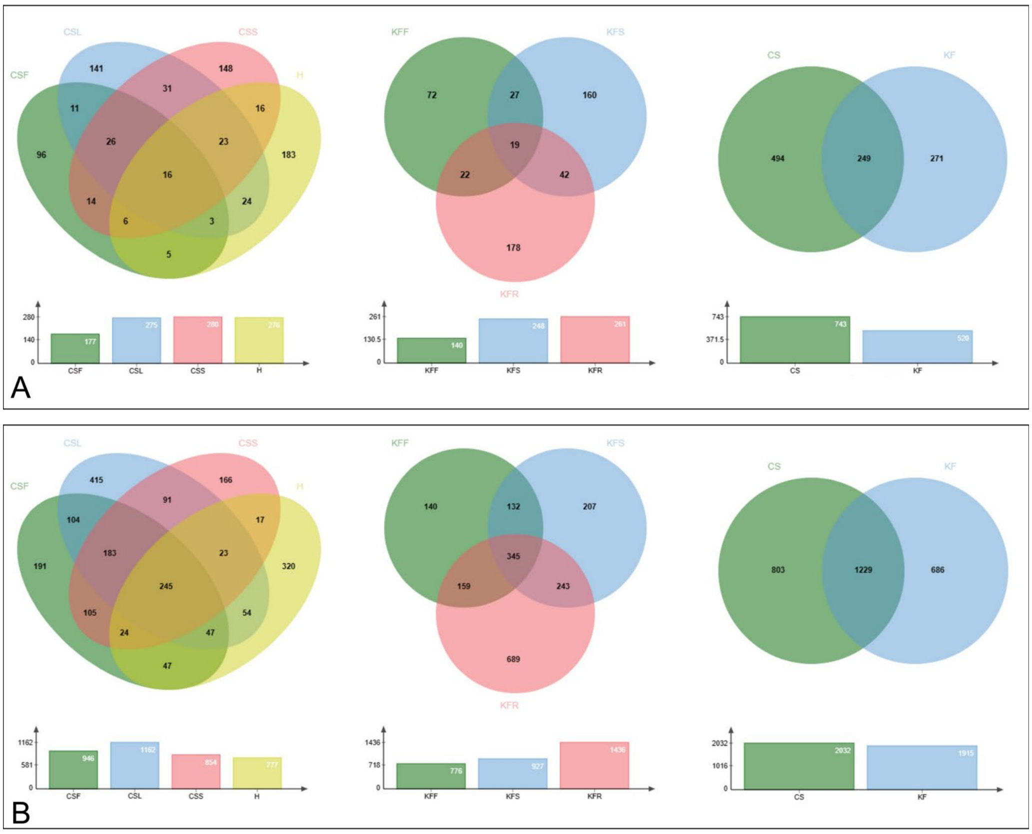

Figure S3. Venn diagram of bacteria (A) and fungi (B).

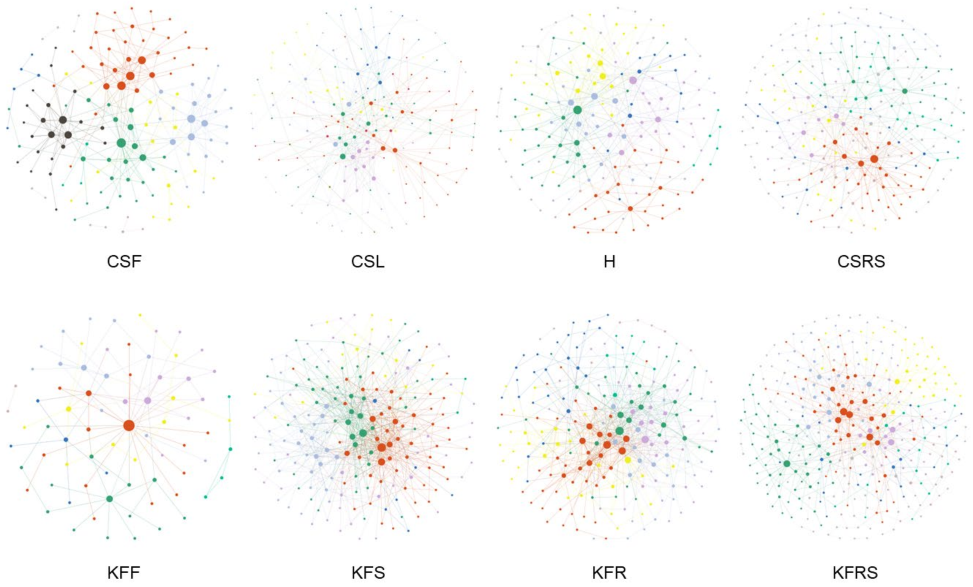

Figure S4. Network analysis of bacteria.

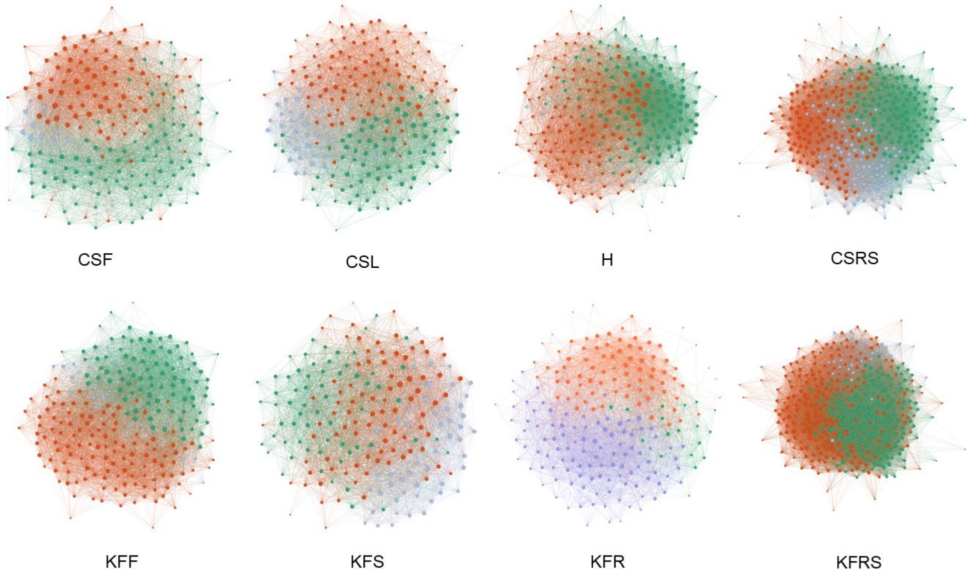

Figure S5. Network analysis of fungi.

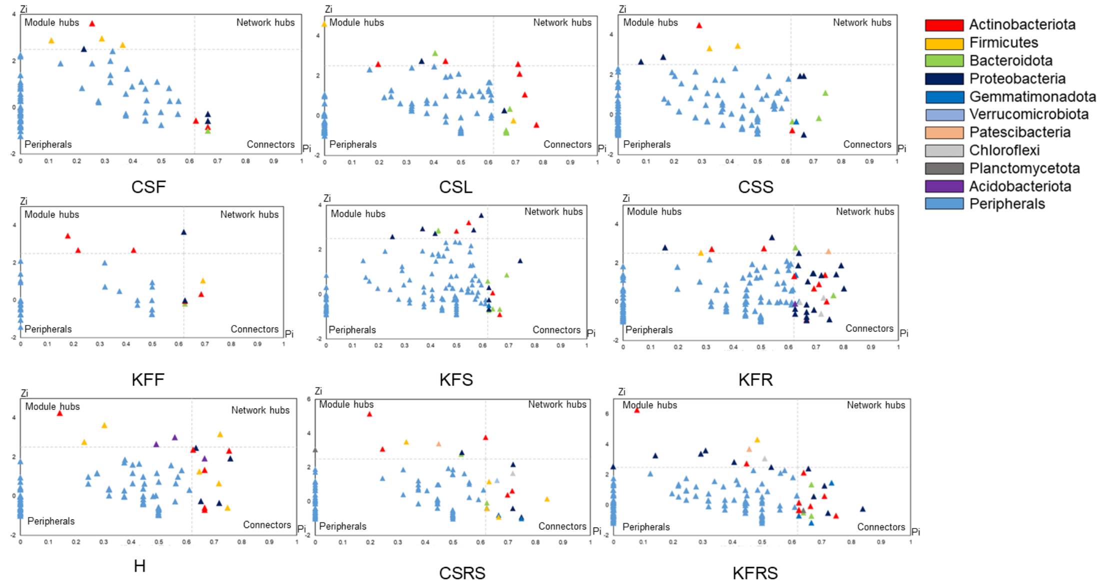

Figure S6. Z-P analysis of bacterial network.

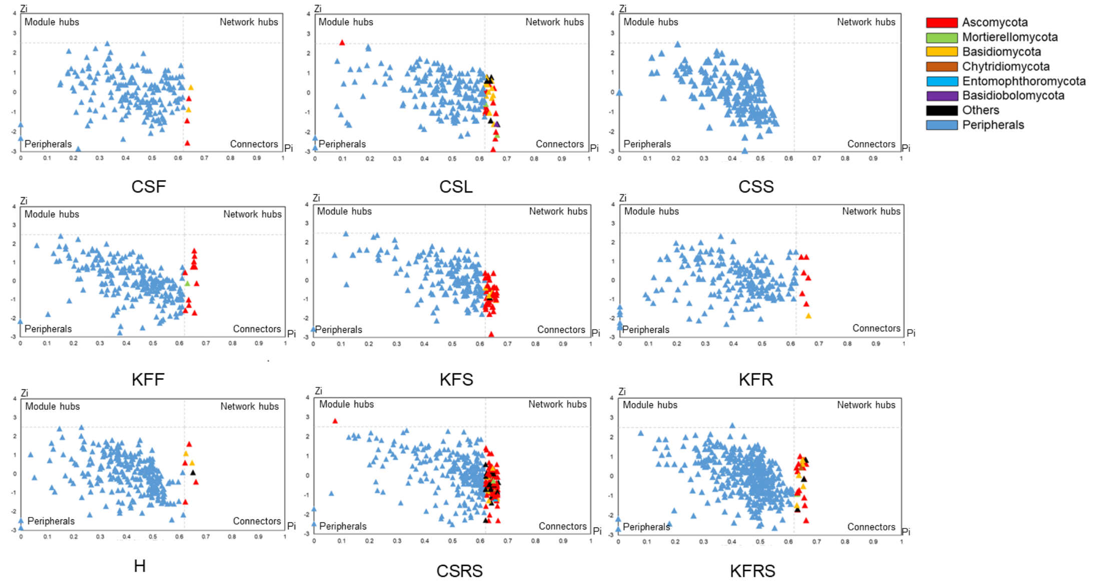

Figure S7. Z-P analysis of fungi network.

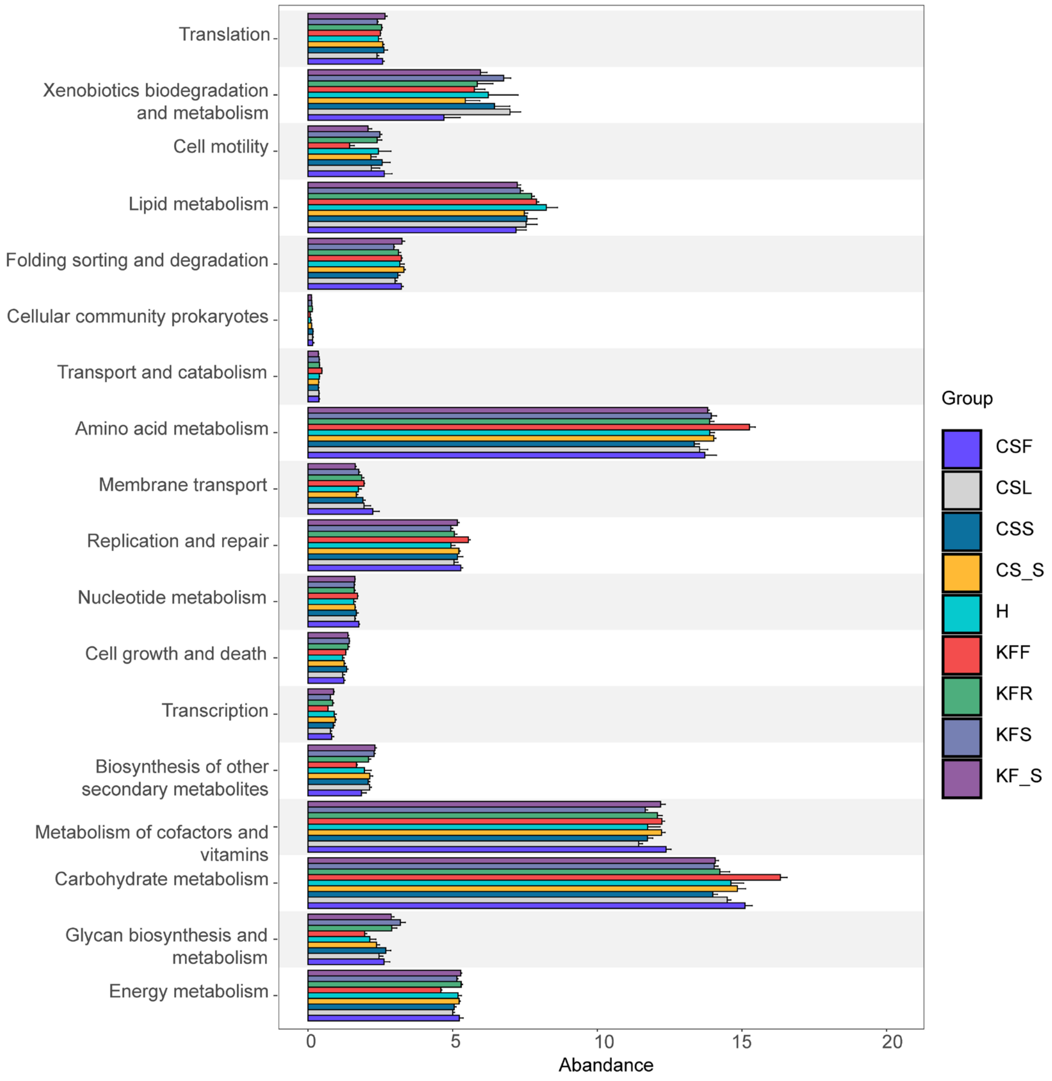

Figure S8. Result of PICRUSt analysis. The p-values of the differential pathways shown in the pictures are all less than 0.5.

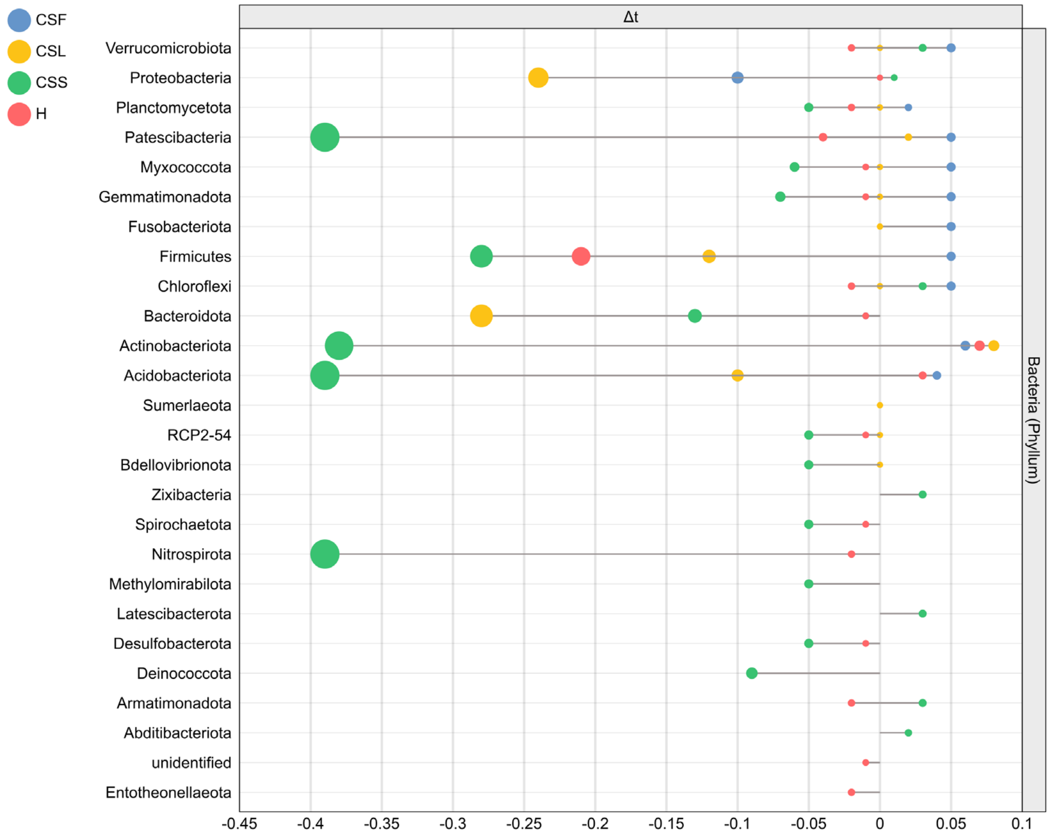

Figure S9. Leave-one-out Procrustes analysis at the level of bacterial phyllum.

Note: Blue represents the flowers of *C. salsa*, yellow represents the scale leaves of *C. salsa*, green represents the fleshy stems of *C. salsa*, and red represents the haustorium of *C. salsa*. The circle size represents the size of |Δt|.

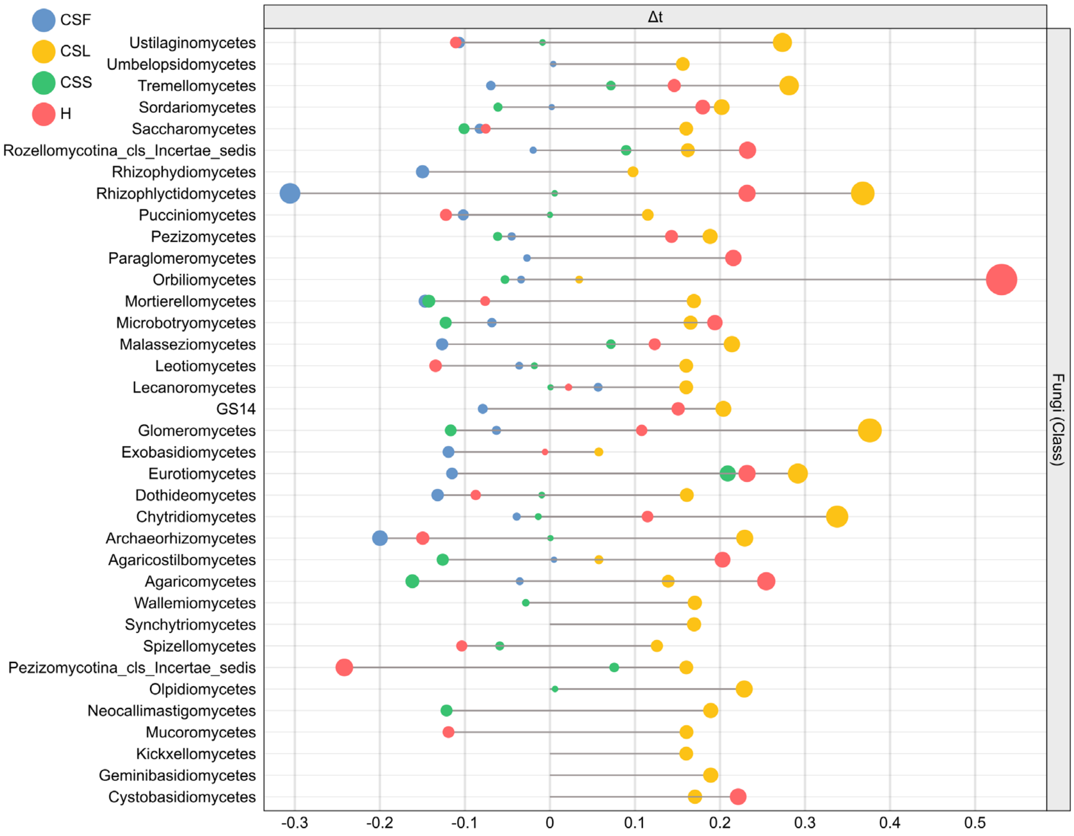

Figure S10. Leave-one-out Procrustes analysis at the level of fungal class.

Note: Blue represents the flowers of *C. salsa*, yellow represents the scale leaves of *C. salsa*, green represents the fleshy stems of *C. salsa*, and red represents the haustorium of *C. salsa*. The circle size represents the size of |Δt|.

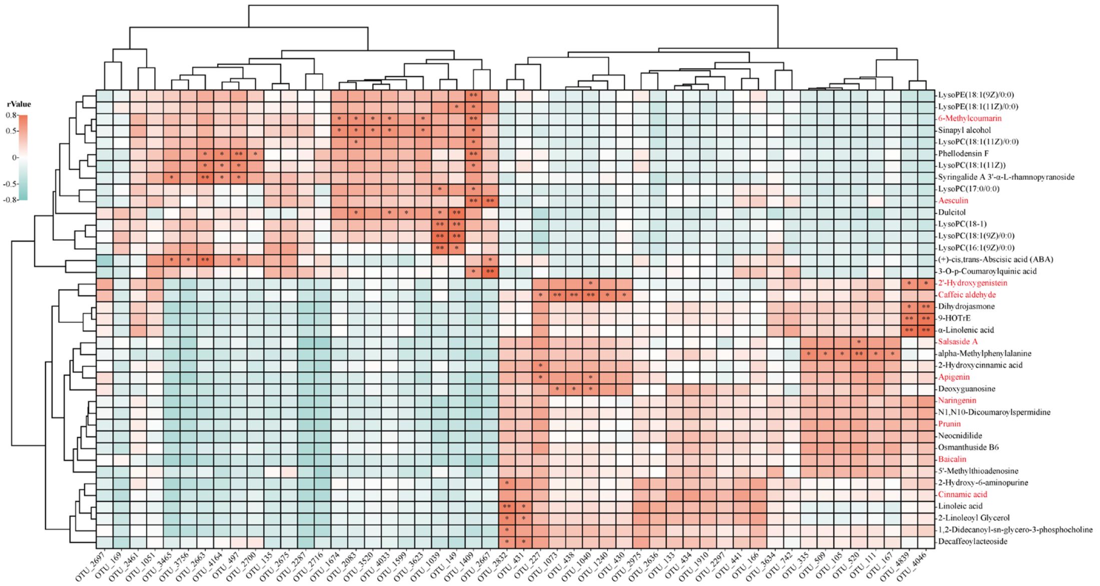

Figure S11. Correlation analysis between differential metabolites of CSF vs CSL and endophytic bacteria of *C. salsa*. Red indicates positive correlation, green indicates negative correlation, and color shades indicate the strength of correlation ("*" indicates $P < 0.05$, "**" indicates $P < 0.01$). The metabolites marked in red are secondary

metabolites.

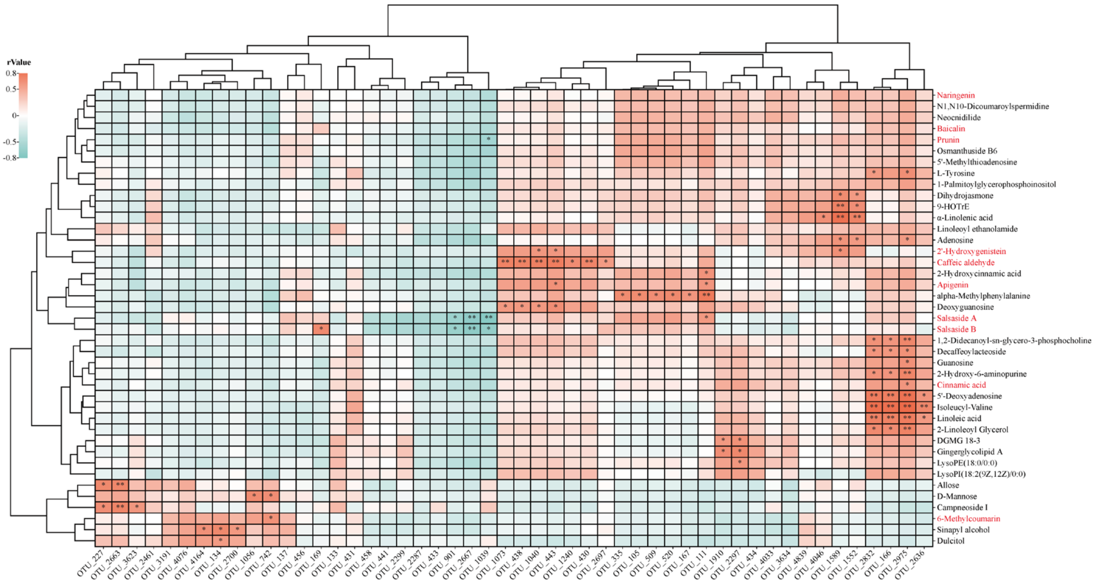

Figure S12. Correlation analysis between differential metabolites of CSF vs CSS and endophytic bacteria of *C. salsa*. Red indicates positive correlation, green indicates negative correlation, and color shades indicate the strength of correlation ("*" indicates $P < 0.05$, "**" indicates $P < 0.01$). The metabolites marked in red are secondary metabolites.

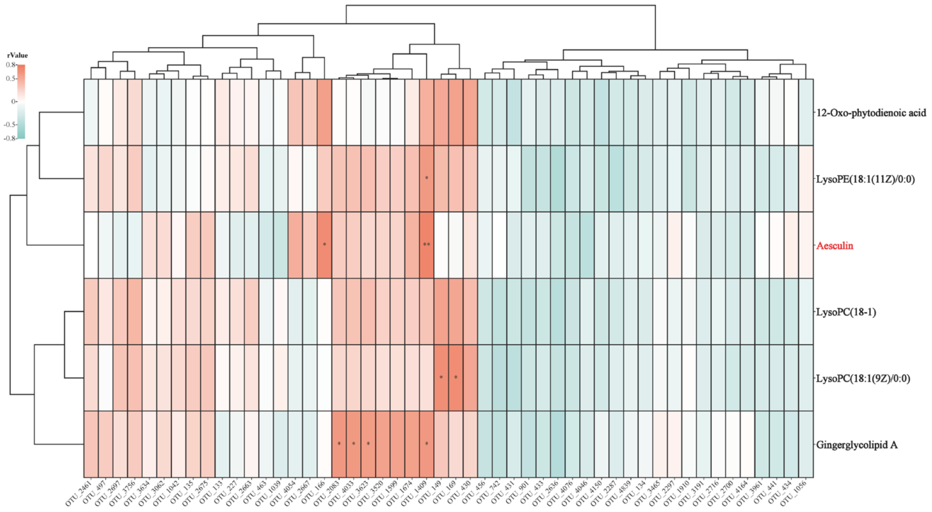

Figure S13. Correlation analysis between differential metabolites of CSS vs CSL and endophytic bacteria of *C. salsa*. Red indicates positive correlation, green indicates negative correlation, and color shades indicate the strength of correlation ("*" indicates $P < 0.05$, "**" indicates $P < 0.01$). The metabolites marked in red are secondary metabolites.

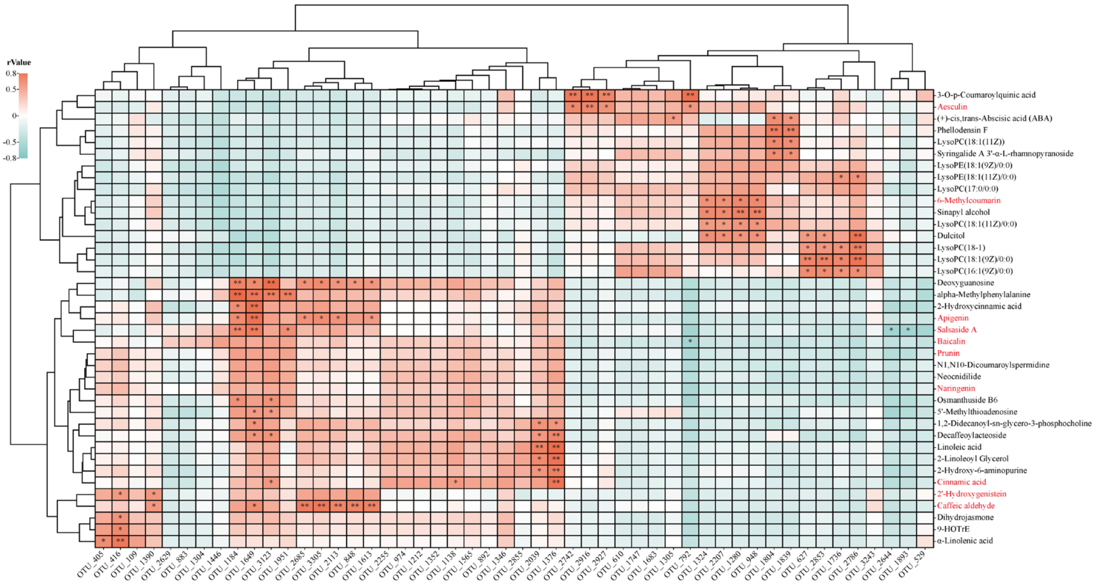

Figure S14. Correlation analysis between differential metabolites of CSF vs CSL and endophytic fungi of *C. salsa*. Red indicates positive correlation, green indicates negative correlation, and color shades indicate the strength of correlation ("*" indicates $P< 0.05$, "**" indicates $P< 0.01$). The metabolites marked in red are secondary metabolites.

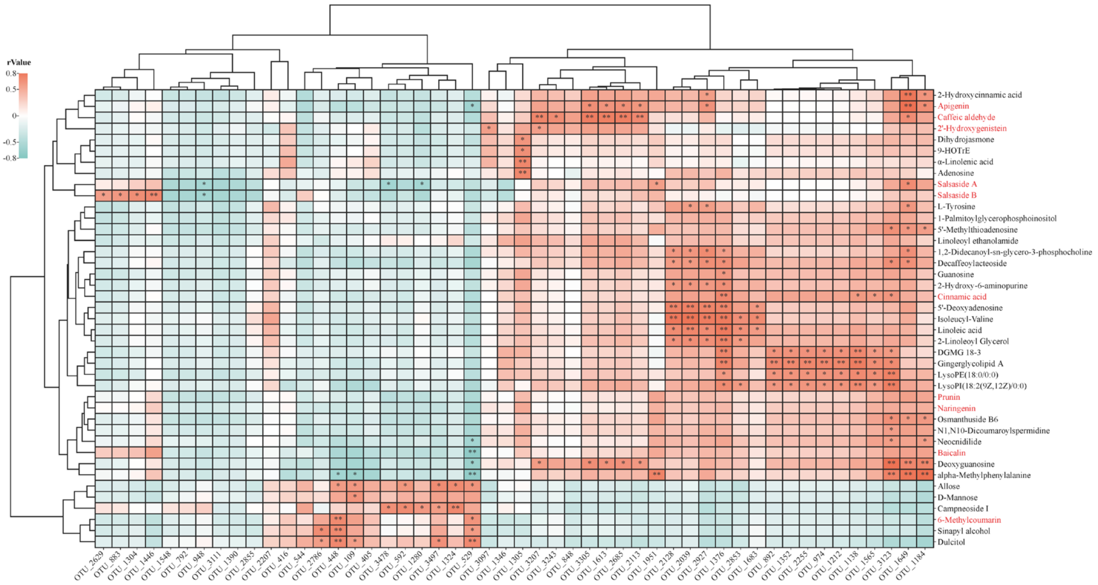

Figure S15. Correlation analysis between differential metabolites of CSF vs CSS and endophytic fungi of *C. salsa*. Red indicates positive correlation, green indicates negative correlation, and color shades indicate the strength of correlation ("*" indicates $P< 0.05$, "**" indicates $P< 0.01$). The metabolites marked in red are secondary metabolites.

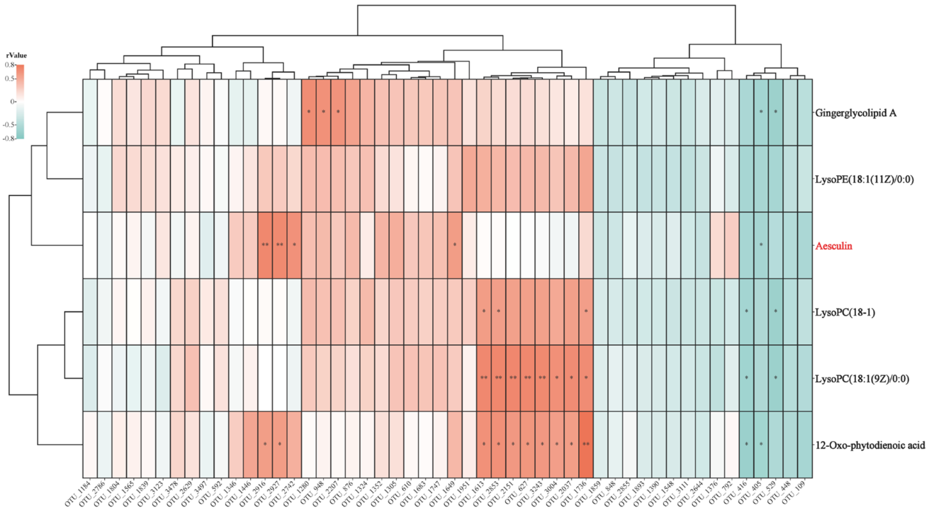

Figure S16. Correlation analysis between differential metabolites of CSF vs CSS and endophytic fungi of *C. salsa*. Red indicates positive correlation, green indicates negative correlation, and color shades indicate the strength of correlation ("*" indicates *P*< 0.05, "**" indicates *P*< 0.01). The metabolites marked in red are secondary metabolites.

Table S1. Classification of the overall metabolic composition of *C. salsa*

Score: Primary mass spectrometry accurate molecular weight matching (20), Secondary mass spectrometry fragment matching (20), Isotope distribution matching (20). Compounds scoring < 36 were considered inaccurately qualified and subsequently removed.

No.	Metabolites	Class	kegg	Score	Formula	CSF	CSL	CSS
1	(-)-Epigallocatechin	Flavonoids	C12136	37	C15H14O7	619.56	579.71	599.74
2	(-)-JA-L-Ile	Carboxylic acids and derivatives	C18699	39	C18H29NO4	572.97	451.28	736.65
3	(-)-Pinoresinol glucoside	Other	-	39	C26H32O11	75.48	6.53	0.00
4	(+)-cis,trans-Abscisic acid (ABA)	Other	-	45	C15H20O4	1808.97	6344.84	3580.44
5	(+)-Lariciresinol 4,4'-bis-O-beta-D-glucopyranoside	Other	-	38	C32H44O16	794.73	1160.07	1100.22
6	(±)-2-Hydroxy-4-(methylthio)butanoic acid	Fatty Acyls	-	38	C5H10O3S	39.78	7.08	9.23
7	(2E,4E)-2,4-Hexadienoic acid	Fatty Acyls	-	41	C6H8O2	540.29	206.94	180.82
8	(2S)-Liquiritigenin	Flavonoids	C09762	40	C15H12O4	48.88	0.00	0.00
9	(R)-Campneoside II	Other	-	39	C29H36O16	2170.39	916.99	1246.84
10	(S)-Abscisic acid	Prenol lipids	C06082	39	C15H20O4	146.37	65.13	75.27
11	(S)-Pinocembrin	Flavonoids	C09827	45	C15H12O4	24.86	0.03	0.00
12	1,2-Didecanoyl-sn-glycero-3-phosphocholine	Other	-	40	C28H56NO8P	4116.93	410.90	310.85
13	1,4-dihydro-1-Methyl-4-oxo-3-pyridinecarboxamide	Pyridines and derivatives	C05843	43	C7H8N2O2	267.57	21.67	20.35
14	10-Hydroxydecanoic acid	Other	-	38	C10H20O3	0.03	1.62	79.74
15	11,12-Epoxyeicosatrienoic acid	Fatty Acyls	-	38	C20H32O3	71.97	0.00	0.00
16	12,13-DiHOME	Fatty Acyls	C14829	41	C18H34O4	295.00	468.99	87.45
17	12,13-EODE	Other	-	42	C18H32O3	1228.21	95.90	6.37
18	12-Oxo-phytodienoic acid	Other	-	41	C18H28O3	10360.44	2525.96	382.29
19	13-HOTrE(r)	Other	-	43	C18H30O3	1177.29	116.49	83.94
20	13-HPODE	Fatty Acyls	C04717	43	C18H32O4	5769.43	459.70	47.54
21	13-HpOTrE(r)	Other	-	40	C18H30O4	332.85	49.34	8.82
22	14,15-Dehydrocrepenynic acid	Fatty Acyls	-	42	C18H28O2	203.80	28.88	0.00
23	157446-63-4.MOL	Other	-	42	C26H34O12	2531.47	4300.24	10194.58
24	1-Heptadecanoylglycerophosphoethanolamine	Glycerophospholipids	-	37	C22H46NO7P	292.13	916.11	336.03
25	1H-Indole-3-carboxaldehyde	Indoles and derivatives	C08493	48	C9H7NO	11941.14	9728.49	6585.25
26	1-Hydroxy-2-naphthoic acid	Other	-	38	C11H8O3	32.16	15.77	26.22
27	1-Methoxyindole-3-carbaldehyde	Other	-	41	C10H9NO2	205.95	331.23	337.77
28	1-Methyladenine	Imidazopyrimidines	C02216	42	C6H7N5	99.61	67.72	88.81
29	1-Methylhistidine	Carboxylic acids and derivatives	C01152	39	C7H11N3O2	0.00	98.45	648.70
30	1-Nonanol	Fatty Acyls	C14696	37	C9H20O	5.11	0.00	0.00
31	1-O-Sinapoyl-beta-D-glucose	Other	-	41	C17H22O10	295.26	555.86	528.75
32	1-Palmitoylglycerophosphoinositol	Glycerophospholipids	-	51	C25H49O12P	20585.74	14364.12	6008.36
33	1-Phenanthrenecarboxylic acid	Other	-	40	C20H26O3	6112.75	4445.78	6047.07
34	2,3-Dihydrobenzofuran	Coumarans	-	46	C8H8O	3160.64	1055.70	1716.67
35	2',4'-Dihydroxyacetophenone	Organooxygen compounds	C03663	42	C8H8O3	35.94	2.13	0.38
36	2'-Deoxyinosine-5'-monophosphate	Other	-	37	C10H13N4O7P	446.61	3038.23	724.60
37	2'-AcetylPoliumoside(Brandioside)	Other	-	40	C37H48O20	154.75	163.57	117.91
38	2-Hydroxy-6-aminopurine	Imidazopyrimidines	-	45	C5H5N5O	9567.33	3030.26	1433.31
39	2-Hydroxyadipic acid	Fatty Acyls	C02360	42	C6H10O5	76.01	62.37	12.05
40	2-Hydroxycinnamic acid	Cinnamic acids and derivatives	C01772	44	C9H8O3	7745.72	3121.21	1996.90
41	2'-Hydroxydaidzein	Isoflavonoids	C02495	38	C15H10O5	63.88	0.00	0.00
42	2'-Hydroxygenistein	Isoflavonoids	C12134	40	C15H10O6	7455.51	71.87	1.15
43	2-Hydroxyphenylacetic acid	Benzene and substituted derivatives	C05852	47	C8H8O3	1438.92	1846.16	1942.99
44	2-Isopropylmalate	Fatty Acyls	C02504	45	C7H12O5	25.70	37.68	7.54
45	2-Linoleoyl Glycerol	Other	-	54	C21H38O4	29279.96	5875.08	3322.46
46	2-Methylglutaric acid	Fatty Acyls	-	39	C6H10O4	208.23	133.60	70.51
47	2-Naphthol	Naphthalenes	C11713	40	C10H8O	50.10	70.00	92.23
48	3-(2-Naphthyl)-D-alanine	Other	-	38	C13H13NO2	10.29	2.75	0.00
49	3-(3-Hydroxyphenyl)propanoic acid	Phenylpropanoic acids	C11457	38	C9H10O3	27.94	222.76	228.04
50	3-(4-Hydroxyphenyl)propionic acid	Phenylpropanoic acids	C01744	44	C9H10O3	231.45	147.28	196.85
51	3,3',4'5-Tetrahydroxystilbene	Stilbenes	C05901	38	C14H12O4	269.79	197.00	157.70
52	3,3'-Di-O-Methylquercetin	Other	-	42	C17H14O7	286.65	140.26	144.04
53	3,4,5-Trimethoxycinnamic acid	Cinnamic acids and derivatives	-	41	C12H14O5	388.68	95.46	26.99
54	3,4-Dihydro-2H-1-benzopyran-2-one	3,4-dihydrocoumarins	C02274	42	C9H8O2	2156.34	922.35	1520.96
55	3,4-Dihydrocoumarin	3,4-dihydrocoumarins	C02274	45	C9H8O2	21.75	609.04	1111.17
56	3,4-Dihydroxybenzeneacetic acid	Phenols	C01161	37	C8H8O4	80.49	336.41	254.95
57	3,4-Dimethoxycinnamic acid	Cinnamic acids and derivatives	-	42	C11H12O4	229.89	874.92	1119.32
58	3,7-Dihydroxy-12-oxocholanoic acid	Steroids and steroid derivatives	-	41	C24H38O5	599.62	180.75	251.32
59	30362-89-1.mol	Other	-	37	C16H23N5O5	63.65	22.41	2.87
60	3-Amino-5-hydroxybenzoic acid	Other	-	41	C7H7NO3	555.59	1369.24	1450.03
61	3-Hydroxy-4-methoxycinnamic acid	Cinnamic acids and derivatives	C10470	48	C10H10O4	42.94	110.99	1136.76
62	3-Hydroxyanthranilic acid	Benzene and substituted derivatives	C00632	39	C7H7NO3	1.94	82.11	71.13
63	3-Hydroxybenzyl alcohol	Benzene and substituted derivatives	C03351	46	C7H8O2	451.90	357.34	185.60
64	3-Hydroxyglutaric acid	Hydroxy acids and derivatives	-	39	C5H8O5	3.91	28.63	75.23
65	3-Methyladenine	Imidazopyrimidines	C00913	38	C6H7N5	165.69	85.55	111.73
66	3-Methylindole	Indoles and derivatives	C08313	37	C9H9N	19.62	25.04	22.07
67	3-Methyl-L-histidine	Carboxylic acids and derivatives	C01152	38	C7H11N3O2	0.00	0.11	26.44
68	3-O-Feruloylquinic acid	Organooxygen compounds	C02572	39	C17H20O9	29.45	57.36	18.29
69	3-O-p-Coumaroylquinic acid	Organooxygen compounds	-	40	C16H18O8	803.37	4790.60	3816.81
70	4-Acetamidobutanoic acid	Carboxylic acids and derivatives	C02946	40	C6H11NO3	50.13	0.00	0.00
71	4-Aminoindole	Other	-	41	C8H8N2	2044.47	2538.36	3270.89
72	4-Hydroxy-3,5-bis(isopropyl)benzaldehyde	Other	-	38	C13H18O2	441.65	311.39	412.15
73	4'-Hydroxy-5,7-dimethoxyflavanone	Other	-	39	C37H40O15	1233.73	2370.48	986.12
74	4-Hydroxybenzaldehyde	Organooxygen compounds	C00633	41	C7H6O2	6686.75	6543.42	3486.00
75	4-Hydroxybenzoic acid	Benzene and substituted derivatives	C00156	40	C7H6O3	1374.40	612.15	599.32
76	4-Hydroxycinnamic acid	Cinnamic acids and derivatives	C00811	43	C9H8O3	1215.89	1724.44	1664.34
77	4-Methoxycinnamic acid	Cinnamic acids and derivatives	-	42	C10H10O3	93.74	8.90	8.72
78	4-Methyl-5-thiazoleethanol	Azoles	C04294	38	C6H9NOS	1432.26	561.17	442.08
79	4-Methylumbelliferone	Coumarins and derivatives	C03081	42	C10H8O3	110.34	121.63	176.78
80	4-oxo-9Z,11Z,13E,15E-octadecatetraenoic acid	Fatty Acyls	-	42	C18H26O3	231.45	118.61	145.02
81	4-Pyridoxic acid	Pyridines and derivatives	C00847	38	C8H9NO4	0.01	9.22	2.00
82	5-Aminolevulinic acid	Carboxylic acids and derivatives	C00430	40	C5H9NO3	1.73	7.70	23.28
83	5'-Deoxy-5'-(methylthio)adenosine	5'-deoxyribonucleosides	C00170	37	C11H15N5O3S	139.05	19.30	26.34
84	5'-Deoxyadenosine	5'-deoxyribonucleosides	C05198	45	C10H13N5O3	3615.24	1441.29	403.07
85	5-Hydroxy-L-tryptophan	Indoles and derivatives	C00643	38	C11H12N2O3	182.60	95.96	100.49
86	5-Hydroxytryptophol	Indoles and derivatives	-	40	C10H11NO2	26.27	0.00	0.00
87	5-Methoxy-N,N-dimethyltryptamine	Indoles and derivatives	C08309	38	C13H18N2O	226.53	150.57	220.61
88	5'-Methylthioadenosine	5'-deoxyribonucleosides	C00170	48	C11H15N5O3S	27500.12	12500.41	7878.86
89	5-Methyluridine	Pyrimidine nucleosides	-	39	C10H14N2O6	475.74	211.11	319.24
90	5-Phenylvaleric acid	Fatty Acyls	-	43	C11H14O2	584.28	538.48	655.20
91	6-Aminocaproic acid	Fatty Acyls	C02378	39	C6H13NO2	23.43	24.50	32.81
92	6-Deoxycatalpol	Other	-	50	C15H22O9	49139.28	52871.56	28694.09
93	6-Hydroxy-4-methylcoumarin	Other	-	46	C10H8O3	38.37	67.87	13.09
94	6-Hydroxymethylherniarin	Other	-	42	C11H10O4	6.13	36.69	71.79
95	6-Methylcoumarin	Coumarins and derivatives	-	43	C10H8O2	1062.93	6806.16	5971.40
96	7,3'-Di-O-Methylquercetin	Polyketides	-	43	C17H14O7	140.50	73.37	6.28
97	8,9-Epoxyeicosatrienoic acid	Fatty Acyls	C14769	37	C20H32O3	73.58	15.82	25.22
98	8-Epiloganic Acid	Other	-	45	C16H24O10	3168.15	4562.74	20998.58
99	9,10-EODE	Other	-	45	C18H32O3	656.01	67.78	0.70
100	932-35-4.mol	Other	-	36	C36H48O20	624.85	326.67	89.16
101	9-Decenoic acid	Fatty Acyls	-	41	C10H18O2	0.00	0.44	9.79
102	9-HOTrE	Fatty Acyls	-	50	C18H30O3	59863.02	5434.67	853.39

103	9-HpOTrE	Fatty Acyls	-	42	C18H30O4	1293.06	592.59	71.88
104	9-KODE	Fatty Acyls	C14766	44	C18H30O3	3347.67	358.47	46.64
105	9-OxoODE	Fatty Acyls	C14766	41	C18H30O3	7450.75	420.12	116.53
106	Adenine	Imidazopyrimidines	C00147	50	C5H5N5	2594.04	1231.30	946.34
107	Adenosine	Purine nucleosides	C00212	51	C10H13N5O4	78009.29	40349.14	28600.49
108	Aesculin	Coumarins and derivatives	C09264	37	C15H16O9	520.51	6495.50	2191.40
109	Ajugol	Other	-	45	C15H24O9	6353.95	7976.85	10211.04
110	Alanylisoleucine	Carboxylic acids and derivatives	-	40	C9H18N2O3	748.82	108.75	13.70
111	Alanylphenylalanine	Carboxylic acids and derivatives	-	37	C12H16N2O3	268.22	97.42	12.62
112	Allose	Organooxygen compounds	C01487	49	C6H12O6	642.17	3811.11	5099.93
113	Allysine(6-Oxo DL-Norleucine)	Other	-	45	C6H11NO3	476.32	160.99	101.87
114	alpha-D-Glucose	Organooxygen compounds	C00267	42	C6H12O6	208.61	1076.83	1615.32
115	Alpha-Linolenic acid	Fatty Acyls	C06427	50	C18H30O2	90.53	99.66	28.34
116	alpha-Methylphenylalanine	Phenylpropanoic acids	-	49	C10H13NO2	31273.29	620.72	209.00
117	A-Monoacylglycerol	Glycerolipids	-	45	C21H36O4	1534.56	1890.00	1101.47
118	Androsterone	Steroids and steroid derivatives	C00523	40	C19H30O2	4536.19	3638.93	3040.32
119	Apigenin	Flavonoids	C01477	51	C15H10O5	133238.79	224.13	24.72
120	Arachidonic acid	Fatty Acyls	C00219	37	C20H32O2	32.58	2.56	0.00
121	Aromadendrin	Flavonoids	C00974	41	C15H12O6	1791.84	108.01	30.63
122	Aspartyl-Isoleucine	Carboxylic acids and derivatives	-	40	C10H18N2O5	3286.48	1677.40	743.69
123	Aspartyl-Tyrosine	Carboxylic acids and derivatives	-	38	C13H16N2O6	1874.52	1690.75	1316.21
124	Asp-phe	Carboxylic acids and derivatives	-	43	C13H16N2O5	25.96	13.83	1.32
125	Azelaic acid	Fatty Acyls	C08261	40	C9H16O4	15.90	0.00	0.00
126	Baicalin	Macrolides and analogues	C10025	41	C21H18O11	82744.77	1120.87	323.30
127	Benzaldehyde	Benzene and substituted derivatives	C00261	43	C7H6O	724.15	356.40	122.93
128	Benzoic acid	Benzene and substituted derivatives	C00539	41	C7H6O2	41.56	11.40	2.27
129	beta-Glycerophosphoric acid	Glycerophospholipids	C02979	40	C3H9O6P	50.67	5.15	4.72
130	Betaine	Carboxylic acids and derivatives	C00719	44	C5H11NO2	48678.10	71378.52	65142.02
131	beta-Lactose	Organooxygen compounds	C01970	37	C12H22O11	86.53	25.14	6.26
132	Betulinic acid	Prenol lipids	C08619	37	C30H48O3	2991.67	260.92	80.56
133	Biliverdin	Tetrapyrroles and derivatives	C00500	40	C33H34N4O6	815.66	491.44	451.31
134	Biocytin	Carboxylic acids and derivatives	C05552	38	C16H28N4O4S	149.08	1.40	0.89
135	Biotin	Biotin and derivatives	C00120	38	C10H16N2O3S	2.55	19.19	8.31
136	B-Monoacylglycerol	Endocannabinoids	C13856	45	C21H36O4	966.43	687.99	385.26
137	Butin	Polyketides	-	45	C15H12O5	165.09	63.99	1.62
138	Butyrylcarnitine	Fatty Acyls	C02862	38	C11H21NO4	17.39	0.08	0.14
139	Cafestol	Naphthofurans	C09066	36	C20H28O3	107.43	64.15	76.33
140	Caffeic acid	Cinnamic acids and derivatives	C01481	57	C9H8O4	768.35	312.99	77.19
141	Caffeic acid O-glucoside	Other	-	50	C15H18O9	2890.63	804.06	185.13
142	Caffeic aldehyde	Cinnamaldehydes	C10945	50	C9H8O3	13791.54	3132.67	2147.26
143	Calceolarioside A	Other	-	44	C23H26O11	358.50	129.65	10.01
144	Calcitriol	Steroids and steroid derivatives	C01673	39	C27H44O3	267.05	0.00	0.00
145	Campneoside I	Other	-	44	C30H38O16	1813.12	3752.24	4715.52
146	Catapol	Other	-	41	C15H22O10	5100.79	9714.71	7191.64
147	Catechin	Flavonoids	C06562	44	C15H14O6	905.34	444.34	276.36
148	Catechol	Phenols	C15571	39	C6H6O2	9.24	0.20	0.25
149	Chlorogenic acid	Organooxygen compounds	C00852	44	C16H18O9	51.64	332.91	77.82
150	Chrysoeriol	Flavonoids	C04293	45	C16H12O6	1481.50	0.00	0.00
151	Cinnamic acid	Cinnamic acids and derivatives	C10438	53	C9H8O2	29752.02	9019.17	5722.29
152	cis-p-Coumaric acid	Cinnamic acids and derivatives	C06738	46	C9H8O3	198.68	478.97	231.23
153	Cistanoside D	Other	-	44	C31H40O15	0.02	123.17	178.74
154	Cistanoside E	Other	-	39	C21H32O12	2095.98	1929.00	1922.01
155	Cistanoside F	Other	-	50	C21H28O13	75787.23	50098.32	36674.75
156	Cistanoside H	Fatty Acyls	-	45	C16H30O2	1814.27	439.40	206.24
157	Cistantubuloside A	Other	-	39	C36H48O19	123.88	229.89	338.74
158	Cistantubuloside C1	Other	-	43	C35H46O21	7686.62	5165.32	5293.28
159	Citric acid	Carboxylic acids and derivatives	C00158	40	C6H8O7	729.19	1598.81	2013.48
160	Cocamidopropyl betaine	Other	-	44	C19H38N2O3	6400.14	5288.76	8557.07
161	Conicaoside	Other	-	43	C27H36O12	229.18	904.92	1612.68
162	Coniferin	Organooxygen compounds	C00761	40	C16H22O8	184.38	768.31	913.44
163	Coniferyl alcohol	Phenols	C00590	48	C10H12O3	16.21	664.86	901.95
164	Coniferylaldehyde	Phenols	C02666	47	C10H10O3	1114.47	2570.59	16409.90
165	Coumarin	Coumarins and derivatives	C05851	40	C9H6O2	2682.79	1396.31	1000.79
166	crenatoside	Other	-	46	C29H34O15	721.53	1149.73	1794.50
167	Cytidine	Pyrimidine nucleosides	C00475	46	C9H13N3O5	0.73	30.23	55.81
168	Cytidine-5'-monophosphate	Pyrimidine nucleotides	C00055	49	C9H14N3O8P	564.37	225.27	51.42
169	D-(+)-Sucrose	Organooxygen compounds	C00089	41	C12H22O11	12.59	148.83	39.47
170	Daphnetin	Coumarins and derivatives	C03093	37	C9H6O4	12.25	6.67	0.00
171	Daucosterol	Other	-	40	C35H60O6	31.02	179.29	301.49
172	Decaffeoylacteoside	Other	-	44	C20H30O12	23012.78	5771.08	3129.58
173	Dehydrophytosphingosine	Organonitrogen compounds	-	55	C18H37NO3	27155.10	34712.00	17931.02
174	Deoxyadenosine	Purine nucleosides	C00559	40	C10H13N5O3	1250.13	488.21	507.15
175	Deoxyguanosine	Purine nucleosides	C00330	40	C10H13N5O4	14983.15	1016.51	401.16
176	Dethiobiotin	Fatty Acyls	C01909	39	C10H18N2O3	130.13	14.69	0.02
177	DGMG 18-3	Other	-	50	C33H56O14	5794.73	2836.82	1218.18
178	Diethylphosphate	Organic phosphoric acids and derivatives	C06608	47	C4H11O4P	729.98	1081.03	1434.42
179	Dihydrojasmone	Organooxygen compounds	-	46	C11H18O	5708.08	686.46	100.67
180	Dihydroquercetin	Other	-	44	C15H12O7	569.26	0.00	0.00
181	Dihydrotestosterone	Steroids and steroid derivatives	C03917	39	C19H30O2	3.07	46.34	0.21
182	Dihydrozeatin (DZ)	Other	-	38	C10H15N5O	759.25	247.75	166.62
183	DL-Norvaline	Carboxylic acids and derivatives	C01799	39	C5H11NO2	22.02	33.02	48.45
184	D-Mannose	Organooxygen compounds	C00936	44	C6H12O6	607.36	10868.69	29360.91
185	Dodecanedioic acid	Fatty Acyls	C02678	44	C12H22O4	581.21	167.22	53.35
186	Dodecanoylcarnitine	Fatty Acyls	-	40	C19H37NO4	500.77	336.02	399.16
187	Dopamine	Phenols	C03758	40	C8H11NO2	744.27	377.70	450.28
188	D-Pantothenic acid	Organooxygen compounds	C00864	40	C9H17NO5	313.35	578.30	577.85
189	Dulcitol	Organooxygen compounds	C01697	41	C6H14O6	2314.22	8738.90	16143.53
190	D-Xylose	Organooxygen compounds	C00181	37	C5H10O5	0.00	5.66	11.32
191	Echinacoside	Other	-	40	C35H46O20	96651.54	102911.27	99759.15
192	Eicosadienoic acid	Fatty Acyls	C16525	41	C20H36O2	1871.08	180.58	157.43
193	Eicosapentaenoic acid	Fatty Acyls	C06428	37	C20H30O2	57.51	11.84	3.72
194	Enterodiol	Dibenzylbutane lignans	C18166	40	C18H22O4	191.27	894.13	383.01
195	Epicatechin	Flavonoids	C09727	38	C15H14O6	1235.18	1359.50	830.15
196	Epimeredinoside A	Other	-	45	C31H40O15	0.00	62.63	95.50
197	Eriojaposide B	Fatty Acyls	-	38	C25H40O11	27.48	0.00	0.00
198	Esculin (6,7-Dihydroxycoumarin-6-glucoside)	Other	-	40	C15H16O9	1297.30	4009.54	3044.96
199	Etiocholanolone	Steroids and steroid derivatives	C04373	37	C19H30O2	130.79	69.50	58.83
200	Eucommin A	Other	-	39	C27H34O12	738.46	554.64	331.95
201	Eutigoside C	Other	-	50	C23H26O9	86.96	243.43	60.83
202	Ferulic acid	Cinnamic acids and derivatives	C01494	51	C10H10O4	0.27	14.78	97.42
203	Formononetin (4'-O-methyldaidzein)	Other	-	39	C16H12O4	144.01	426.00	186.28
204	Formononetin 7-O-glucoside (ononin)	Other	-	38	C22H22O9	1298.03	1627.69	1331.94
205	Fraxin	Other	-	39	C16H18O10	112.09	386.77	271.14
206	Galactitol	Organooxygen compounds	C01697	40	C6H14O6	113.88	126.78	85.30
207	Gallic acid	Benzene and substituted derivatives	C01424	45	C7H6O5	29.46	0.43	0.00
208	gamma-Glutamylleucine	Carboxylic acids and derivatives	-	39	C11H20N2O5	1574.87	2936.23	2555.11
209	Gamma-Linolenic acid	Fatty Acyls	C06426	40	C18H30O2	201.65	297.39	43.18

210	Geniposidic acid	Prenol lipids	C11673	49	C16H22O10	3.75	42.17	61.16
211	Genistein	Isoflavonoids	C06563	41	C15H10O5	35.90	0.00	0.00
212	Gibberellin A20	Prenol lipids	C02035	40	C19H24O5	68.63	15.26	29.19
213	Gibberellin A4 (GA4)	Other	-	43	C19H24O5	4.85	60.83	122.82
214	Gibberellin A9	Prenol lipids	C11863	38	C19H24O4	115.08	18.27	31.67
215	Gingerglycolipid A	Glycerolipids	-	52	C33H56O14	5756.19	3086.51	758.73
216	Gingerglycolipid B	Glycerolipids	-	53	C33H58O14	32800.11	11268.08	7548.03
217	Glutaminylisoleucine	Carboxylic acids and derivatives	-	38	C11H21N3O4	676.27	110.22	29.53
218	Glutamylleucine	Carboxylic acids and derivatives	-	42	C11H20N2O5	2823.79	724.03	120.54
219	Glycitin	Isoflavonoids	-	42	C22H22O10	2191.20	1819.62	2169.49
220	Glycylleucine	Carboxylic acids and derivatives	C02155	40	C8H16N2O3	368.90	44.66	10.17
221	Guanine	Imidazopyrimidines	C00242	38	C5H5N5O	125.48	79.25	70.26
222	Guanosine	Purine nucleosides	C00387	46	C10H13N5O5	7238.35	3201.67	1573.09
223	Guanosine 3′,5′-cyclic monophosphate	Other	-	42	C10H11N5O7P-	0.00	165.78	0.25
224	Hesperetin	Flavonoids	C01709	45	C16H14O6	325.41	5.29	0.00
225	Hesperetin 5-O-glucoside	Flavonoids	-	37	C22H24O11	3.64	237.68	68.41
226	Hexadecanedioic acid	Fatty Acyls	C19615	41	C16H30O4	255.13	38.29	4.13
227	Histidinol	Organonitrogen compounds	C00860	39	C6H11N3O	135.12	326.20	196.38
228	Homogentisic acid	Benzene and substituted derivatives	C00544	45	C8H8O4	596.42	1838.97	1075.48
229	Homovanillic acid	Phenols	C05582	51	C9H10O4	416.26	382.84	170.41
230	Hordatine B	2-arylbenzofuran flavonoids	C08308	38	C29H40N8O5	436.73	39.26	0.00
231	Hordenine	Benzene and substituted derivatives	C06199	41	C10H15NO	119.29	27.99	8.78
232	Icariin	Polyketides	-	43	C33H40O15	0.36	52.80	54.93
233	Indole	Indoles and derivatives	C00463	46	C8H7N	2472.77	2332.96	1321.38
234	Indole-2-carboxylic acid	Indoles and derivatives	-	38	C9H7NO2	7.68	13.37	24.94
235	Indole-3-propionic acid	Indoles and derivatives	-	40	C11H11NO2	95.22	10.75	2.07
236	Indoleacrylic acid	Indoles and derivatives	-	46	C11H9NO2	134666.21	114581.15	80828.83
237	Indolepyruvate	Indoles and derivatives	C00331	38	C11H9NO3	5.67	13.42	16.25
238	Inosine	Purine nucleosides	C00294	41	C10H12N4O5	2700.43	1932.86	1218.18
239	Isoacteoside	Cinnamic acids and derivatives	-	51	C29H36O15	34676.49	41800.39	30167.20
240	Isobutyryl-L-carnitine	Fatty Acyls	-	38	C11H21NO4	5.06	74.00	18.85
241	Isoleucyl-Valine	Carboxylic acids and derivatives	-	41	C11H22N2O3	3775.45	772.20	194.48
242	Isonicotinic acid	Pyridines and derivatives	C07446	41	C6H5NO2	1786.59	803.01	578.76
243	Isoquinoline	Isoquinolines and derivatives	C06323	41	C9H7N	673.71	597.43	405.68
244	Isorhamnetin	Flavonoids	C10084	42	C16H12O7	254.00	495.93	202.68
245	Isorhamnetin 3-(6''-malonylglucoside)	Flavonoids	-	38	C25H24O15	1246.01	26.65	24.83
246	Isosakuranetin (4'-Methylnaringenin)	Other	-	39	C16H14O5	139.91	72.33	23.10
247	Itaconic acid	Fatty Acyls	C00490	38	C5H6O4	0.84	63.66	13.63
248	Jionoside A1	Other	-	45	C36H48O20	9698.42	9346.56	10131.70
249	Jionoside C	Other	-	38	C29H36O13	76.77	304.56	242.52
250	Kaempferide	Flavonoids	C10098	38	C16H12O6	1139.73	1070.21	982.41
251	Kaempferol 3-O-rutinoside (nicotiflorin)	Other	-	38	C27H30O15	342.80	445.18	277.63
252	Kankanose	Other	-	40	C28H40O18	1823.43	2689.90	1434.49
253	Kankanoside A	Other	-	44	C16H26O8	3414.83	45.79	0.78
254	Kankanoside B	Other	-	39	C15H24O10	40.05	134.12	95.04
255	Kankanoside E	Other	-	41	C16H28O8	3.81	227.90	85.58
256	Kankanoside F	Other	-	37	C26H40O17	545.15	976.29	257.73
257	Kankanoside G	Other	-	40	C30H38O14	2634.98	531.44	859.91
258	Kankanoside H1	Other	-	47	C37H48O20	10689.38	5378.52	5447.52
259	Kankanoside H2	Other	-	44	C38H50O20	325.94	0.65	0.20
260	Kankanoside L	Other	-	39	C15H24O9	65.08	93.53	114.90
261	Kankanoside O	Other	-	43	C16H26O8	2085.21	871.71	1274.60
262	Kankanoside P	Other	-	44	C16H26O8	3032.60	109.39	68.10
263	L-(-)-Tyrosine	Other	-	41	C9H11NO3	5056.66	2675.53	2422.86
264	l-(+)-Arginine	Carboxylic acids and derivatives	C00062	41	C6H14N4O2	17.80	320.11	686.22
265	L-(+)-Lysine	Carboxylic acids and derivatives	C00047	42	C6H14N2O2	54.00	0.00	0.00
266	L-3-Phenyllactic acid	Phenylpropanoic acids	C05607	43	C9H10O3	1007.69	604.37	871.50
267	L-Alloisoleucine	Carboxylic acids and derivatives	-	41	C6H13NO2	59.79	1.54	0.00
268	L-Arginine	Carboxylic acids and derivatives	C00062	40	C6H14N4O2	19.51	24.66	135.73
269	Lauric acid (C12-0)	Other	-	40	C12H24O2	986.50	1221.29	739.67
270	L-Carnitine	Organonitrogen compounds	C00318	38	C7H15NO3	15.08	0.00	0.00
271	L-Cysteine	Carboxylic acids and derivatives	C00097	39	C3H7NO2S	27.62	27.59	33.77
272	Leucyl-Leucine	Carboxylic acids and derivatives	C11332	40	C12H24N2O3	2259.20	377.23	137.63
273	L-Glutamic acid	Carboxylic acids and derivatives	C00025	43	C5H9NO4	2481.30	775.60	859.42
274	Linoleic acid	Fatty Acyls	C01595	47	C18H32O2	7416.11	1323.96	791.29
275	Linoleoyl ethanolamide	Organonitrogen compounds	-	42	C20H37NO2	16897.65	9981.29	5007.15
276	L-Isoleucine	Carboxylic acids and derivatives	C00407	47	C6H13NO2	4346.14	3087.01	3252.11
277	L-Leucine	Carboxylic acids and derivatives	C00123	56	C6H13NO2	5998.29	4266.19	4087.34
278	L-Methionine methyl ester	Other	-	41	C6H13NO2S	47.17	27.17	23.42
279	L-Norleucine	Carboxylic acids and derivatives	C01933	40	C6H13NO2	5.71	17.14	10.91
280	L-Phenylalanine	Carboxylic acids and derivatives	C00079	45	C9H11NO2	101.72	50.79	66.27
281	L-Proline	Carboxylic acids and derivatives	C00148	42	C5H9NO2	116.07	63.32	77.95
282	L-Saccharopine	Carboxylic acids and derivatives	C00449	38	C11H20N2O6	81.97	119.98	90.08
283	L-Tyrosine	Carboxylic acids and derivatives	C00082	43	C9H11NO3	11913.37	6379.70	4416.80
284	Lugrandoside	Other	-	38	C29H36O16	7.03	72.88	18.34
285	Lumichrome	Other	-	37	C12H10N4O2	2.60	2.37	15.05
286	Luteolin	Flavonoids	C01514	41	C15H10O6	56.81	3.78	3.26
287	LysoPC 17-2	Other	-	41	C25H48NO7P	1280.57	690.34	410.84
288	LysoPC(0:0/16:0)	Glycerophospholipids	-	52	C24H50NO7P	225137.09	335019.43	173076.59
289	LysoPC(0:0/18:2(9Z,12Z))	Glycerophospholipids	-	54	C26H50NO7P	173350.72	93079.22	68350.84
290	LysoPC(14:0/0:0)	Glycerophospholipids	-	47	C22H46NO7P	4052.53	4305.44	1956.64
291	LysoPC(15:0/0:0)	Glycerophospholipids	C04230	42	C23H48NO7P	1449.64	3115.45	1217.71
292	LysoPC(16:1(9Z)/0:0)	Glycerophospholipids	C04230	44	C24H48NO7P	935.52	10368.28	2494.95
293	LysoPC(17:0/0:0)	Glycerophospholipids	C04230	48	C25H52NO7P	2254.46	8258.61	2938.08
294	LysoPC(18:0)	Glycerophospholipids	C04230	41	C26H54NO7P	2797.93	1765.36	1317.71
295	LysoPC(18:0/0:0)	Glycerophospholipids	C04230	48	C26H54NO7P	57663.65	53939.10	19229.81
296	LysoPC(18:1(11Z))	Glycerophospholipids	C04230	50	C26H52NO7P	10184.11	51491.15	18254.96
297	LysoPC(18:1(11Z)/0:0)	Glycerophospholipids	C04230	52	C26H52NO7P	9724.47	104888.17	37550.15
298	LysoPC(18:1(9Z)/0:0)	Glycerophospholipids	-	57	C26H52NO7P	5544.95	175779.58	39832.65
299	LysoPC(18:2(9Z,12Z))	Glycerophospholipids	-	50	C26H50NO7P	189492.46	233946.96	153177.47
300	LysoPC(18:2(9Z,12Z)/0:0)	Glycerophospholipids	-	41	C26H50NO7P	6820.48	4059.44	2950.45
301	LysoPC(18:3(6Z,9Z,12Z)/0:0)	Glycerophospholipids	C04230	52	C26H48NO7P	6764.88	15106.21	6870.10
302	LysoPC(18:3(9Z,12Z,15Z))	Glycerophospholipids	-	42	C26H48NO7P	6162.06	7714.04	2730.68
303	LysoPC(18-1)	Other	-	54	C26H52NO7P	994.81	10941.60	3264.92
304	LysoPC(18-3)	Other	-	37	C26H48NO7P	278.62	219.34	112.23
305	LysoPC(18-4)	Other	-	41	C26H46NO7P	266.55	78.31	2.54
306	LysoPC(20:2(11Z,14Z)/0:0)	Glycerophospholipids	C04230	44	C28H54NO7P	8618.31	6832.18	3347.69
307	LysoPC(20-3(5Z,8Z,11Z))	Other	-	38	C28H52NO7P	109.34	200.31	25.35
308	LysoPC(20-5)	Other	-	42	C28H48NO7P	5.77	288.83	2.42
309	LysoPC(22-1)	Other	-	36	C30H60NO7P	1.17	23.97	27.63
310	LysoPC(24-1)	Other	-	47	C32H64NO7P	36.08	66.72	10.12
311	LysoPE(0:0/16:0)	Glycerophospholipids	-	47	C21H44NO7P	2249.55	2462.92	2428.94
312	LysoPE(0:0/16:1(9Z))	Glycerophospholipids	-	42	C21H42NO7P	111.64	734.59	75.48
313	LysoPE(15:0/0:0)	Glycerophospholipids	-	42	C20H42NO7P	279.89	540.27	208.04
314	LysoPE(16:0/0:0)	Glycerophospholipids	-	56	C21H44NO7P	41803.73	52205.17	30082.89
315	LysoPE(16:1(9Z)/0:0)	Glycerophospholipids	-	40	C21H42NO7P	1404.45	34.41	0.55
316	LysoPE(18:0/0:0)	Glycerophospholipids	-	42	C23H48NO7P	5192.34	3958.93	1375.69

317	LysoPE(18:1(11Z)/0:0)	Glycerophospholipids	-	50	C23H46NO7P	2740.61	37633.42	14489.64
318	LysoPE(18:1(9Z)/0:0)	Glycerophospholipids	-	43	C23H46NO7P	1539.27	5795.12	2327.86
319	LysoPE(18:2(9Z,12Z)/0:0)	Glycerophospholipids	-	57	C23H44NO7P	63720.74	30745.57	16296.68
320	LysoPE(20:2(11Z,14Z)/0:0)	Glycerophospholipids	-	41	C25H48NO7P	614.42	24.04	14.59
321	LysoPG(18:2(9Z,12Z)/0:0)	Glycerophospholipids	-	49	C24H45O9P	6053.20	3140.32	2024.26
322	LysoPI(18:2(9Z,12Z)/0:0)	Glycerophospholipids	-	53	C27H49O12P	31649.17	11360.59	5506.24
323	LysoPS(18:1(9Z)/0:0)	Glycerophospholipids	-	43	C24H46NO9P	4.85	82.18	54.94
324	LysoPS(18:2(9Z,12Z)/0:0)	Glycerophospholipids	-	39	C24H44NO9P	4056.22	2723.70	2964.45
325	Lysylleucine	Carboxylic acids and derivatives	-	38	C12H25N3O3	1284.89	267.76	76.11
326	Lysylphenylalanine	Carboxylic acids and derivatives	-	37	C15H23N3O3	983.36	161.59	57.21
327	MAG (18-1)	Other	-	49	C21H40O4	173.21	142.79	11.62
328	MAG 18-4	Other	-	42	C21H34O4	174.86	25.31	21.37
329	MAG 18-5	Other	-	43	C21H32O4	286.82	878.30	841.80
330	Maltotriose	Organooxygen compounds	C01835	40	C18H32O16	1295.02	754.32	2064.80
331	Mandelic acid	Benzene and substituted derivatives	C01984	44	C8H8O3	1046.30	552.54	328.48
332	Mannitol	Organooxygen compounds	C00392	42	C6H14O6	48.21	7.26	2.67
333	m-Coumaric acid	Cinnamic acids and derivatives	C12621	41	C9H8O3	91.97	110.36	160.10
334	Methoxyindoleacetic acid	Indoles and derivatives	C05660	42	C11H11NO3	204.68	549.41	311.36
335	Methyl indole-3-acetate	Indoles and derivatives	-	38	C11H11NO2	0.00	74.19	94.43
336	Methyl vanillate	Benzene and substituted derivatives	-	37	C9H10O4	45.41	2.86	1.92
337	Methyldopa	Phenylpropanoic acids	C07194	39	C10H13NO4	521.80	123.02	59.09
338	Methyleugenol	Benzene and substituted derivatives	C10454	44	C11H14O2	273.57	332.05	366.53
339	Methylglutaric acid	Fatty Acyls	-	40	C6H10O4	195.07	93.92	34.18
340	Mevalonolactone	Lactones	-	40	C6H10O3	7.46	24.97	8.39
341	MG(0:0/14:0/0:0)	Glycerolipids	-	40	C17H34O4	276.70	1.70	5.52
342	MG(0:0/18:2(9Z,12Z)/0:0)	Fatty Acyls	-	49	C21H38O4	4823.62	1256.16	958.92
343	MG(18:1(9Z)/0:0/0:0)	Glycerolipids	-	52	C21H40O4	296.91	589.96	176.05
344	MG(18:3(9Z,12Z,15Z)/0:0/0:0)	Fatty Acyls	-	43	C21H36O4	5016.28	3545.18	1657.77
345	MGMG 18-2	Other	-	50	C27H48O9	5968.05	2187.91	1511.91
346	Morin	Flavonoids	C10105	39	C15H10O7	552.52	0.23	1.85
347	Mussaenoside	Other	-	44	C17H26O10	954.29	1919.41	1286.53
348	Myristoleic acid	Fatty Acyls	C08322	41	C14H26O2	66.34	38.46	23.31
349	N-(1-Deoxy-1-fructosyl)isoleucine	Carboxylic acids and derivatives	-	38	C12H23NO7	4563.65	4057.18	4622.50
350	N-(1-Deoxy-1-fructosyl)phenylalanine	Carboxylic acids and derivatives	-	39	C15H21NO7	5035.76	2381.83	1465.33
351	N-(3-Methylbut-2-EN-1-YL)-9H-purin-6-amine	Other	-	39	C10H13N5	154.90	107.83	156.78
352	N1,N10-Dicoumaroylspermidine	Cinnamic acids and derivatives	-	51	C25H31N3O4	58131.83	2412.50	1447.04
353	N1-Acetylspermidine	Carboximidic acids and derivatives	C00612	39	C9H21N3O	424.84	852.04	927.35
354	N2-methylguanosine	Other	-	43	C11H15N5O5	3077.83	2081.76	1541.43
355	N-Acetyl-L-glutamic acid	Carboxylic acids and derivatives	C00624	41	C7H11NO5	26.29	12.71	15.06
356	N-Acetyl-l-leucine	Carboxylic acids and derivatives	C02710	39	C8H15NO3	51.72	144.86	95.80
357	N-Acetylproline	Carboxylic acids and derivatives	-	40	C7H11NO3	972.94	45.95	39.70
358	NAD	(5'->5')-dinucleotides	C00003	39	C21H27N7O14P2	367.99	109.71	60.90
359	N-alpha-Acetyl-L-lysine	Carboxylic acids and derivatives	C12989	38	C8H16N2O3	13.24	0.00	0.00
360	Naringenin	Flavonoids	C00509	46	C15H12O5	52783.44	2239.76	1137.34
361	Naringenin chalcone	Linear 1,3-diarylpropanoids	C06561	43	C15H12O5	84.44	7.72	0.00
362	Naringin	Flavonoids	C09789	39	C27H32O14	5252.10	1131.17	801.57
363	Neocnidilide	Isobenzofurans	C17002	40	C12H18O2	8458.80	3011.09	2115.53
364	N'-Formylkynurenine	Organooxygen compounds	C02406	38	C11H12N2O4	134.25	22.18	20.27
365	N-Glycyl-L-leucine	Carboxylic acids and derivatives	C02155	37	C8H16N2O3	20.39	0.21	0.31
366	Nicotinate ribonucleoside	Organooxygen compounds	C05841	42	C11H13NO6	151.41	165.61	147.53
367	N-Lauryldiethanolamine	Other	-	45	C16H35NO2	57677.66	102933.50	120109.33
368	Nobiletin	Flavonoids	C10112	40	C21H22O8	43.53	213.36	187.37
369	Normetanephrine	Phenols	C05589	38	C9H13NO3	57.36	2.49	1.02
370	N-Palmitoyl Serine	Fatty Acyls	-	39	C19H37NO4	544.37	39.15	52.15
371	N-p-coumaroyl-N'-caffeoylputrescine	Other	-	39	C22H24N2O5	250.18	175.20	77.28
372	Octadeca-11E,13E,15Z-trienoic acid	Fatty Acyls	-	44	C18H30O2	126.71	62.31	1.63
373	O-Desmethylangolensin	Alpha-methyldeoxybenzoin flavonoids	-	38	C15H14O4	77.94	84.47	107.51
374	Oleic acid	Fatty Acyls	C00712	41	C18H34O2	173.00	315.98	72.83
375	O-Phosphocholine	Organonitrogen compounds	C00588	40	C5H15NO4P+	9609.63	9066.56	4995.33
376	osmanthuside B	Cinnamic acids and derivatives	-	39	C29H36O13	479.02	202.46	250.19
377	Osmanthuside B6	Other	-	44	C29H36O13	31148.67	9928.99	5891.44
378	Oxidized-adrenal-ferredoxin	Phenols	-	39	C9H13NO3	190.78	239.14	108.14
379	Palatinose	Other	-	47	C12H22O11	19854.56	24845.25	29227.38
380	Palmitoylethanolamide	Carboximidic acids and derivatives	C16512	45	C18H37NO2	602.10	189.85	92.83
381	Pantetheine	Carboxylic acids and derivatives	C00831	37	C11H22N2O4S	162.41	154.09	174.05
382	Pantothenic acid	Organooxygen compounds	C00864	42	C9H17NO5	11729.87	12178.89	10433.38
383	Pantothenol	Organooxygen compounds	C05944	39	C9H19NO4	0.00	17.57	4.86
384	p-Coumaraldehyde	Cinnamaldehydes	C05608	43	C9H8O2	5286.18	5126.33	2750.42
385	p-Coumaric acid	Cinnamic acids and derivatives	C00811	38	C9H8O3	99.92	31.75	45.75
386	Pentylbenzene	Benzene and substituted derivatives	-	40	C11H16	91.64	0.00	0.00
387	Phellodensin F	Polyketides	-	41	C26H30O10	708.95	10669.77	21056.27
388	Phenylacetaldehyde	Benzene and substituted derivatives	C00601	44	C8H8O	2209.65	520.37	306.17
389	Phenylalanyl-Glutamate	Carboxylic acids and derivatives	-	40	C14H18N2O5	1717.66	507.80	163.31
390	Phenylalanyl-Valine	Carboxylic acids and derivatives	-	39	C14H20N2O3	1243.87	416.92	296.68
391	Phenylephrine	Phenols	C07441	41	C9H13NO2	890.05	1376.03	1726.73
392	Phenyllactic acid	Phenylpropanoic acids	C01479	37	C9H10O3	538.46	826.56	785.67
393	phenylsuccinic acid	Other	-	41	C10H10O4	323.13	2057.39	1769.69
394	Phe-Phe	Carboxylic acids and derivatives	-	38	C18H20N2O3	97.01	0.00	0.00
395	Phloretin	Linear 1,3-diarylpropanoids	C00774	39	C15H14O5	239.91	223.21	94.26
396	Phthalic acid	Benzene and substituted derivatives	C01606	49	C8H6O4	2404.95	1425.24	1921.87
397	Phthalic anhydride	Other	-	40	C8H4O3	670.84	655.84	878.44
398	Phytocassane D	Prenol lipids	-	39	C20H28O3	101.43	3.42	0.76
399	Phytosphingosine	Organonitrogen compounds	C12144	53	C18H39NO3	621.47	179.28	48.81
400	Pinoresinol	Other	-	40	C20H22O6	172.77	442.61	1087.09
401	Pinoresinol di-O-beta-D-glucopyranoside	Other	-	40	C32H42O16	1188.84	708.18	955.52
402	Piperidine	Piperidines	C01746	42	C5H11N	16.74	6.56	7.22
403	Plantainoside A	Other	-	40	C23H26O11	2467.85	2332.38	687.83
404	Plantainoside B	Other	-	47	C23H26O11	17936.50	29177.66	22022.91
405	Plantainoside C	Other	-	38	C30H38O15	582.70	1710.18	2072.47
406	Plantainoside D	Other	-	42	C29H36O16	509.95	1136.37	1496.37
407	p-Mentha-1,3,5,8-tetraene	Benzene and substituted derivatives	-	42	C10H12	7.18	0.00	0.00
408	Propionylcarnitine	Fatty Acyls	C03017	41	C10H19NO4	25.72	0.78	0.82
409	Protocatechuic aldehyde	Organooxygen compounds	C16700	37	C7H6O3	33.97	31.61	41.39
410	Prunin	Polyketides	-	48	C21H22O10	15300.31	3560.25	2761.03
411	p-Synephrine	Phenols	C04548	39	C9H13NO2	0.57	121.71	0.59
412	Punicic acid	Fatty Acyls	-	47	C18H30O2	221.26	643.50	164.62
413	Quercetin 3-O-glucoside (isotrifoliin)	Other	-	40	C21H20O12	162.02	17.23	1.25
414	Quinine	Cinchona alkaloids	C06526	38	C20H24N2O2	68.08	6.91	2.81
415	Resveratrol	Stilbenes	C03582	39	C14H12O3	41.59	16.98	10.54
416	Riboflavin	Pteridines and derivatives	C00255	40	C17H20N4O6	182.80	158.61	1216.35
417	sakuranetin	Flavonoids	C09833	42	C16H14O5	580.18	168.37	200.53
418	Salicylic acid	Benzene and substituted derivatives	C00805	43	C7H6O3	238.28	181.69	274.52
419	Salicylic acid (SA)	Other	-	41	C7H6O3	661.95	312.09	191.03
420	Salsaside A	Other	-	44	C28H34O13	8466.87	3675.66	3664.83
421	Salsaside B	Other	-	38	C28H34O13	14323.50	9200.38	6393.79
422	Salsaside C	Other	-	47	C28H34O12	64.68	64.72	32.56
423	Salsaside D	Other	-	42	C31H38O15	1144.69	142.31	22.64

424	Salsaside F	Other	-	44	C31H38O15	900.90	1146.17	494.91
425	Scopoletin	Coumarins and derivatives	C01752	40	C10H8O4	359.59	203.00	272.33
426	Scopoletin (7-Hydroxy-5-methoxycoumarin)	Other	-	49	C10H8O4	474.08	432.26	612.83
427	Sebacate	Fatty Acyls	C08277	36	C10H16O4-2	189.89	88.80	71.93
428	Sebacic acid	Fatty Acyls	C08277	41	C10H18O4	274.70	31.10	41.02
429	Serotonin	Indoles and derivatives	C00780	39	C10H12N2O	60.97	34.52	0.00
430	Serylleucine	Carboxylic acids and derivatives	-	42	C9H18N2O4	2184.58	549.14	138.68
431	Seryltyrosine	Carboxylic acids and derivatives	-	37	C12H16N2O5	1525.56	2572.18	2331.91
432	Sinapic acid	Cinnamic acids and derivatives	C00482	43	C11H12O5	677.60	3800.08	2824.03
433	Sinapinaldehyde	Other	-	40	C11H12O4	126.84	767.50	1026.94
434	Sinapoyl aldehyde	Other	-	52	C11H12O4	397.57	795.77	524.13
435	Sinapoyl malate	Other	-	41	C15H16O9	167.08	157.11	13.32
436	Sinapyl alcohol	Phenols	C02325	43	C11H14O4	1286.26	11797.21	8204.10
437	sn-Glycero-3-phosphocholine	Glycerophospholipids	C00670	39	C8H20NO6P	199.32	135.50	92.81
438	Sorbitol	Organooxygen compounds	C00794	41	C6H14O6	757.94	1562.76	1591.35
439	Spermine	Organonitrogen compounds	C00750	40	C10H26N4	19.73	48.14	68.56
440	Sphinganine	Organonitrogen compounds	C00836	47	C18H39NO2	2600.01	2455.46	1247.10
441	Stearidonic acid	Fatty Acyls	C16300	39	C18H28O2	120.99	0.05	0.00
442	Styrene	Benzene and substituted derivatives	C19506	40	C8H8	77.66	7.24	0.00
443	Suberic acid	Fatty Acyls	C08278	37	C8H14O4	172.44	281.28	615.23
444	Succinyladenosine	Purine nucleosides	-	45	C14H17N5O8	6248.20	3569.31	2659.70
445	Sumiki's acid	Furans	C20448	39	C6H6O4	0.12	26.11	220.01
446	Syringaldehyde	Other	-	39	C9H10O4	12.83	14.66	9.31
447	Syringalide A 3'-α-L-rhamnopyranoside	Other	-	39	C29H36O14	3958.71	12677.86	20822.17
448	Syringalide A-3'-Rhamnoside	Other	-	36	C29H36O14	187.98	304.12	100.71
449	Syringaresinol	Furanoid lignans	-	39	C22H26O8	544.66	1307.82	3094.38
450	Syringic acid	Benzene and substituted derivatives	C10833	40	C9H10O5	95.36	337.53	313.04
451	Syringin	Other	-	38	C17H24O9	31.50	0.00	0.00
452	Tangeretin	Polyketides	-	40	C20H20O7	146.50	61.45	67.50
453	Terephthalic acid	Benzene and substituted derivatives	C06337	43	C8H6O4	1297.50	781.09	1083.34
454	Tetrahydrodeoxycortisol	Steroids and steroid derivatives	C14594	39	C21H34O4	1622.86	1.25	0.00
455	Thiamine	Diazines	C00378	41	C12H17N4OS+	4410.22	270.33	127.12
456	Threonylisoleucine	Carboxylic acids and derivatives	-	41	C10H20N2O4	1502.50	351.06	44.91
457	Toringin	Flavonoids	-	42	C21H20O9	156.92	27.42	37.48
458	trans-5-O-(4-coumaroyl)-D-quinic acid	Organooxygen compounds	-	52	C16H18O8	67.02	117.54	90.58
459	trans-cinnamaldehyde	Cinnamaldehydes	C00903	46	C9H8O	422.47	2225.80	1698.91
460	trans-Cinnamic acid	Cinnamic acids and derivatives	C10438	42	C9H8O2	24.37	424.50	564.99
461	trans-Ferulic acid	Cinnamic acids and derivatives	C01494	42	C10H10O4	45.10	206.03	1324.72
462	Trans-Hexa-dec-2-enoic acid	Fatty Acyls	-	38	C16H30O2	620.92	16.24	13.15
463	Traumatic acid	Fatty Acyls	C16308	40	C12H20O4	1708.05	1018.49	568.23
464	Trifolin	Flavonoids	-	45	C21H20O11	439.08	1519.24	695.14
465	Trigonelline	Other	C01004	39	C7H7NO2	798.73	146.88	218.70
466	TRIPHENYL PHOSPHATE	Other	-	40	C18H15O4P	1346.27	772.32	896.31
467	Tryptophol	Indoles and derivatives	C00955	44	C10H11NO	7164.12	6007.99	4085.50
468	Tubuloside A	Other	-	40	C37H48O21	10.12	63.46	728.90
469	Tubuloside B	Other	-	40	C31H38O16	276.30	53.37	41.99
470	Tyramine	Benzene and substituted derivatives	C00483	46	C8H11NO	22.78	17.09	13.40
471	Tyrosyl-Leucine	Carboxylic acids and derivatives	-	37	C15H22N2O4	1449.41	355.14	83.61
472	Ubiquinone-1	Prenol lipids	C00399	40	C14H18O4	366.18	738.64	517.10
473	Umbelliferone	Coumarins and derivatives	C09315	44	C9H6O3	22479.56	14633.75	8941.96
474	Undecylenic acid	Fatty Acyls	C13910	43	C11H20O2	699.15	136.12	24.63
475	Uric acid	Imidazopyrimidines	C00366	40	C5H4N4O3	52.53	82.01	57.73
476	Urocanic acid	Azoles	C00785	40	C6H6N2O2	6.37	1.65	2.61
477	Valylalanine	Carboxylic acids and derivatives	-	42	C8H16N2O3	164.82	1.94	0.00
478	Valylleucine	Carboxylic acids and derivatives	-	40	C11H22N2O3	967.97	150.62	11.09
479	Valyl-Leucine	Carboxylic acids and derivatives	-	40	C11H22N2O3	3277.56	541.65	81.61
480	Valylphenylalanine	Carboxylic acids and derivatives	-	39	C14H20N2O3	985.59	418.23	525.86
481	Valylthreonine	Carboxylic acids and derivatives	-	37	C9H18N2O4	452.84	102.11	34.69
482	Valylvaline	Carboxylic acids and derivatives	-	42	C10H20N2O3	70.61	9.61	0.00
483	Vanillic acid	Benzene and substituted derivatives	C06672	41	C8H8O4	2080.25	14935.12	6149.90
484	Vanillin	Phenols	C00755	46	C8H8O3	1340.70	502.92	282.03
485	Verbascoside	Cinnamic acids and derivatives	C10501	42	C29H36O15	89623.43	62198.20	48343.23
486	Vitamin D3	Steroids and steroid derivatives	C05443	39	C27H44O	554.80	143.03	130.84
487	Warfarin	Coumarins and derivatives	C01541	38	C19H16O4	0.00	22.66	0.00
488	Xanthohumol	Linear 1,3-diarylpropanoids	C16417	39	C21H22O5	185.34	640.00	295.75
489	Xanthosine	Purine nucleosides	C01762	43	C10H12N4O6	40.50	8.40	23.55
490	α-Linolenic acid	Fatty Acyls	C06427	44	C18H30O2	13811.26	1430.50	430.21
491	β-Pseudouridine	Nucleoside and nucleotide analogues	C02067	40	C9H12N2O6	756.48	562.55	801.27

Table S2. Significantly different metabolites of different parts of *C. salsa*

Group	Compunds	Class	log2（FC）	P value	VIP
	Apigenin	Phenylpropanoids and polyketides	-9.22	0	7.19
	LysoPC（18:1（9Z）/0:0）	Lipids and lipid-like molecules	4.99	0.01	6.61
	Baicalin	Phenylpropanoids and polyketides	-6.21	0	5.65
	LysoPC（18:1（11Z）/0:0）	Lipids and lipid-like molecules	3.43	0	5.32
	N1,N10-Dicoumaroylspermidine	Phenylpropanoids and polyketides	-4.59	0	4.78
	Naringenin	Phenylpropanoids and polyketides	-4.56	0	4.49
	9-HOTrE	Lipids and lipid-like molecules	-3.46	0.01	4.2
	alpha-Methylphenylalanine	Phenylpropanoids and polyketides	-5.66	0	3.31
	LysoPE（18:1（11Z）/0:0）	Lipids and lipid-like molecules	3.78	0	3.29
	LysoPC（18:1（11Z））	Lipids and lipid-like molecules	2.34	0.02	3.28
	2-Linoleoyl Glycerol	Others	-2.32	0.02	2.86
	Osmanthuside B6	Others	-1.65	0	2.82
	Cinnamic acid	Phenylpropanoids and polyketides	-1.72	0.02	2.72
	Decaffeoylacteoside	Others	-2	0.02	2.41
	5'-Methylthioadenosine	Nucleosides, nucleotides, and analogues	-1.14	0.04	2.27
	Deoxyguanosine	Nucleosides, nucleotides, and analogues	-3.88	0	2.23
	Prunin	Lipids and lipid-like molecules	-2.1	0	2.16
	α-Linolenic acid	Lipids and lipid-like molecules	-3.27	0.04	1.9
	Caffeic aldehyde	Phenylpropanoids and polyketides	-2.14	0.02	1.82
CSF vs CSL	Sinapyl alcohol	Benzenoids	3.2	0.01	1.73
	Phellodensin F	Lipids and lipid-like molecules	3.91	0.02	1.69
	LysoPC（18-1）	Lipids and lipid-like molecules	3.46	0.01	1.65
	Syringalide A 3'-α-L-rhamnopyranoside	Others	1.68	0.01	1.62
	2-Hydroxy-6-aminopurine	Organoheterocyclic compounds	-1.66	0.03	1.55
	LysoPC（16:1（9Z）/0:0）	Lipids and lipid-like molecules	3.47	0.02	1.51
	Neocnidilide	Organoheterocyclic compounds	-1.49	0	1.48
	Linoleic acid	Lipids and lipid-like molecules	-2.49	0.03	1.44
	2'-Hydroxygenistein	Phenylpropanoids and polyketides	-6.7	0.04	1.39
	Dihydrojasmone	Organic oxygen compounds	-3.06	0.01	1.32
	Dulcitol	Organic oxygen compounds	1.92	0.02	1.31
	6-Methylcoumarin	Phenylpropanoids and polyketides	2.68	0	1.29
	Aesculin	Phenylpropanoids and polyketides	3.64	0.01	1.29
	2-Hydroxycinnamic acid	Phenylpropanoids and polyketides	-1.31	0.02	1.29
	Salsaside A	Others	-1.2	0	1.27
	1,2-Didecanoyl-sn-glycero-3-phosphocholine	Others	-3.33	0	1.23
	LysoPC（17:0/0:0）	Lipids and lipid-like molecules	1.87	0.01	1.2
	3-O-p-Coumaroylquinic acid	Organic oxygen compounds	2.58	0.01	1.04
	LysoPE（18:1（9Z）/0:0）	Lipids and lipid-like molecules	1.91	0.01	1.04
	（+）-cis,trans-Abscisic acid	Others	1.81	0.05	1.02
	Apigenin	Phenylpropanoids and polyketides	-12.4	0	6.98
	Baicalin	Phenylpropanoids and polyketides	-8	0	5.6
	N1,N10-Dicoumaroylspermidine	Phenylpropanoids and polyketides	-5.33	0	4.72
	Naringenin	Phenylpropanoids and polyketides	-5.54	0	4.47
	9-HOTrE	Lipids and lipid-like molecules	-6.13	0.01	4.47
	Adenosine	Nucleosides, nucleotides, and analogues	-1.45	0.02	3.71
	D-Mannose	Organic oxygen compounds	5.6	0.01	3.27
	alpha-Methylphenylalanine	Phenylpropanoids and polyketides	-7.23	0	3.19
	Osmanthuside B6	Others	-2.4	0	2.98
	2-Linoleoyl Glycerol	Others	-3.14	0.01	2.95
	Cinnamic acid	Phenylpropanoids and polyketides	-2.38	0.01	2.86
	LysoPI（18:2（9Z,12Z）/0:0）	Lipids and lipid-like molecules	-2.52	0.03	2.72
	Decaffeoylacteoside	Others	-2.88	0	2.62
	5'-Methylthioadenosine	Nucleosides, nucleotides, and analogues	-1.8	0.01	2.46
	Dulcitol	Organic oxygen compounds	2.8	0	2.27
	Deoxyguanosine	Nucleosides, nucleotides, and analogues	-5.22	0	2.22
	1-Palmitoylglycerophosphoinositol	Lipids and lipid-like molecules	-1.78	0.01	2.14
	Prunin	Lipids and lipid-like molecules	-2.47	0	2.13
	α-Linolenic acid	Lipids and lipid-like molecules	-5.01	0.03	2.03
	Caffeic aldehyde	Phenylpropanoids and polyketides	-2.68	0.01	1.9
CSF vs CSS	Linoleoyl ethanolamide	Organic nitrogen compounds	-1.76	0.04	1.69
	2-Hydroxy-6-aminopurine	Organoheterocyclic compounds	-2.74	0.01	1.68
	Neocnidilide	Organoheterocyclic compounds	-2	0	1.58
	Linoleic acid	Lipids and lipid-like molecules	-3.23	0.02	1.46
	L-Tyrosine	Organic acids and derivatives	-1.43	0.03	1.45
	2'-Hydroxygenistein	Phenylpropanoids and polyketides	-12.66	0.04	1.44
	Sinapyl alcohol	Benzenoids	2.67	0.03	1.43
	Dihydrojasmone	Organic oxygen compounds	-5.83	0	1.41
	Guanosine	Nucleosides, nucleotides, and analogues	-2.2	0.01	1.37
	2-Hydroxycinnamic acid	Phenylpropanoids and polyketides	-1.96	0.01	1.34
	Allose	Organic oxygen compounds	2.99	0.03	1.27
	6-Methylcoumarin	Phenylpropanoids and polyketides	2.49	0.02	1.24
	Gingerglycolipid A	Lipids and lipid-like molecules	-2.92	0.02	1.21
	1,2-Didecanoyl-sn-glycero-3-phosphocholine	Others	-3.73	0	1.19
	DGMG 18-3	Others	-2.25	0.03	1.13
	Salsaside B	Others	-1.16	0.01	1.13
	Salsaside A	Others	-1.21	0.01	1.06
	Campneoside I	Others	0.04	0.14	1.05

	Isoleucyl-Valine	Organic acids and derivatives	-4.28	0.04	1.05
	5'-Deoxyadenosine	Nucleosides, nucleotides, and analogues	-3.17	0.01	1.05
	LysoPE（18:0/0:0）	Lipids and lipid-like molecules	-1.92	0.02	1.03
CSL vs CSS	LysoPC（18:1（9Z）/0:0）	Lipids and lipid-like molecules	-2.14	0.05	9.51
	LysoPE（18:1（11Z）/0:0）	Lipids and lipid-like molecules	-1.38	0.05	3.49
	LysoPC（18-1）	Lipids and lipid-like molecules	-1.74	0.04	2.23
	Aesculin	Phenylpropanoids and polyketides	-1.57	0.05	1.54
	Gingerglycolipid A	Lipids and lipid-like molecules	-2.02	0.02	1.17
	12-Oxo-phytodienoic acid	Others	-2.72	0.04	1.13

Table S3. Results of Procrustes analyses.

Classification	Group	Bacteria		Fungi	
		t_0	*p*	t_0	*p*
Parasitic plant	CSF-CSL	0.38	0.74	0.6	0.3
	CSF-CSS	0.77	0.07	0.21	0.82
	CSF-H	0.49	0.58	0.37	0.57
	CSL-CSS	0.47	0.7	0.45	0.44
	CSL-H	0.4	0.73	0.5	0.38
	CSS-H	0.61	0.3	0.91	0.01
Parasitic plant vs Host Plant	CSF-KFF	0.52	0.47	0.66	0.06
	CSF-KFR	0.52	0.49	0.52	0.5
	CSF-KFS	0.77	0.04	0.61	0.3
	CSL-KFF	0.39	0.82	0.57	0.29
	CSL-KFR	0.44	0.36	0.46	0.7
	CSL-KFS	0.85	0.02	0.48	0.64
	CSS-KFF	0.62	0.3	0.24	0.95
	CSS-KFR	0.55	0.3	0.53	0.44
	CSS-KFS	0.44	0.73	0.68	0.17
	H-KFF	0.35	0.85	0.37	0.65
	H-KFR	0.55	0.2	0.46	0.64
	H-KFS	0.36	0.83	0.63	0.2
Host Plant	KFF-KFR	0.38	0.88	0.41	0.58
	KFF-KFS	0.46	0.71	0.1	0.98
	KFS-KFR	0.47	0.44	0.69	0.13
Parasitic plant vs Rhizosphere	CSS-CS-S	0.36	0.83	0.47	0.47
	CSF-CS-S	0.5	0.45	0.79	0.07
	CSL-CS-S	0.23	0.79	0.2	0.93
	H-CS-S	0.76	0.13	0.39	0.49
Host plant vs Rhizosphere	KFR-KF-S	0.36	0.37	0.18	0.99
	KFS-KF-S	0.52	0.32	0.66	0.2
	KFF-KF-S	0.71	0.04	0.16	0.99
Haustorium vs Host plant’s rhizosphere	H-KF-S	0.81	0.05	0.8	0.03

Table S4. Taxonomic information on endophytes associated with the accumulation of secondary metabolites. Abbreviations: CSF: Flower of *C. salsa*; CSL: Leaf of C. salsa; CSS: Stem of *C. salsa*; H: Haustorium of *C. salsa;* KFR: Root of *K. foliatum*; The latter groups are consistently compared to the former, for instance, "CSF-CSL" denotes CSL compared to CSF. Compounds labeled in green signify depletion, those in red represent enrichment, and microorganisms labeled in blue indicate a negative correlation with the corresponding compound.

Group	Compound	Bacteria		Fungi	
		phylum	genus	phylum	genus
CSF-CSL	Apigenin	Firmicutes	Bacillus	Ascomycotag	Unidentified
		Firmicutes	Planomicrobium	Ascomycota	Neocamarosporium
		/	/	Basidiomycota	Cryptococcus
		/	/	Basidiomycota	Naganishia
		/	/	Ascomycota	Alternaria
		/	/	Unidentified	Unidentified
	Caffeic aldehyde	Firmicutes	Planomicrobium	Basidiomycota	Cryptococcus
		Firmicutes	Desemzia	Basidiomycota	Naganishia
		Firmicutes	Planomicrobium	Unidentified	Unidentified
		Firmicutes	Planococcus	Ascomycota	Alternaria
		Firmicutes	Staphylococcus	Ascomycota	Alternaria
		Firmicutes	Bacillus	Ascomycotag	Unidentified
		/	/	Ascomycota	Dimorphosporicola
	2'-Hydroxygenistein	Firmicutes	Clostridium	Ascomycota	Dimorphosporicola
		Firmicutes	Terrisporobacter	Basidiomycota	Unidentified
		Firmicutes	Planomicrobium	/	/
	6-Methylcoumarin	Bacteroidota	Hymenobacter	Ascomycota	Alternaria
		Bacteroidota	Rufibacter	Ascomycota	Dematiopleospora
		Bacteroidota	Hymenobacter	Mortierellomycota	Unidentified
		Proteobacteria	Rubellimicrobium	Ascomycota	Unidentified
		Proteobacteria	Altererythrobacter	/	/
		Proteobacteria	Paracoccus	/	/
	Aesculin	Actinobacteriota	Nocardioides	Basidiomycota	Unidentified
		Bacteroidota	Hymenobacter	Ascomycota	Laburnicola
		/	/	Unidentified	Unidentified
		/	/	Unidentified	Unidentified
	Salsaside A	Proteobacteria	Pantoea	Ascomycota	Unidentified
		/	/	Ascomycota	Neocamarosporium
		/	/	Ascomycota	Alternaria
		/	/	Ascomycota	Pseudogymnoascus
		/	/	Ascomycota	Aspergillus
	Baicalin	/	/	Unidentified	Unidentified
	Naringenin	/	/	Unidentified	Unidentified
	Cinnamic acid	/	/	Ascomycota	Neocamarosporium
		/	/	Ascomycota	Neodidymelliopsis
		/	/	Ascomycota	Unidentified
CSF-CSS	Apigenin	Firmicutes	Terribacillus	Ascomycota	Unidentified
		Firmicutes	Bacillus	Ascomycota	Neocamarosporium
		/	/	Basidiomycota	Cryptococcus
		/	/	Basidiomycota	Naganishia
		/	/	Unidentified	Unidentified
		/	/	Unidentified	Unidentified
		/	/	Ascomycota	Alternaria
		/	/	Basidiobolomycota	Unidentified
	Cinnamic acid	Fusobacteriota	Cetobacterium	Ascomycota	Neocamarosporium
		/	/	Ascomycota	Neodidymelliopsis
		/	/	Ascomycota	Unidentified
		/	/	Ascomycota	Unidentified
	Prunin	Actinobacteriota	Rubrobacter	/	/
	Caffeic aldehyde	Firmicutes	Terribacillus	Basidiomycota	Cryptococcus
		Firmicutes	Planomicrobium	Basidiomycota	Naganishia
		Firmicutes	Desemzia	Unidentified	Unidentified
		Firmicutes	Planomicrobium	Ascomycota	Unidentified
		Firmicutes	Staphylococcus	Ascomycota	Alternaria
		Firmicutes	Planococcus	Ascomycota	Didymella
		Actinobacteriota	Kocuria	Ascomycota	Unidentified
	2'-Hydroxygenistein	Bacteroidota	Prevotella	Ascomycota	Penidiella

		Firmicutes	Planomicrobium	Ascomycota	Unidentified
		Firmicutes	Terribacillus	/	/
	6-Methylcoumarin	Firmicutes	Mycoplasma	Unidentified	Unidentified
		/	/	Basidiobolomycota	Unidentified
	Salsaside B	Actinobacteriota	Nocardioides	Ascomycota	Unidentified
		Proteobacteria	Pantoea	Ascomycota	Unidentified
		Actinobacteriota	Rubrobacter	Ascomycota	Aureobasidium
		Proteobacteria	Enterobacter	Ascomycota	Dematiopleospora
		/	/	Unidentified	Unidentified
	Salsaside A	Actinobacteriota	Rubrobacter	Ascomycota	Unidentified
		Actinobacteriota	Nocardioides	Basidiomycota	Trichosporon
		Proteobacteria	Enterobacter	Ascomycota	Alternaria
		Firmicutes	Bacillus	Ascomycota	Dematiopleospora
		/	/	Ascomycota	Alternaria
	Baicalin	/	/	Basidiobolomycota	Unidentified
	Aesculin	Bacteroidota	Hymenobacter	Basidiomycota	Basidiomycota
		Firmicutes	Bacillus	Unidentified	Unidentified
CSS-CSL		/	/	Ascomycota	Laburnicola
		/	/	Ascomycota	Ascomycota
		/	/	Basidiomycota	Calostoma
